# Sesamin attenuates atherosclerosis by alleviating vascular endothelial ferroptosis-related injury *via* m^6^A-dependent regulation of SREBF1 expression

**DOI:** 10.3389/fcell.2026.1807359

**Published:** 2026-06-23

**Authors:** Xiaoying Chen, Guanyi Zheng, Kailong Fu, Minglong Yao, Kan Lin

**Affiliations:** Department of Traditional Chinese Medicine, Fujian Medical University Affiliated Union Hospital, Fuzhou, Fujian, China

**Keywords:** atherosclerosis, ferroptosis, M6A, sesamin, SREBF1, Yishen Huoxue Huatan formula

## Abstract

**Objective:**

This study was designed to elucidate the molecular pathways responsible for anti-atherosclerotic effects of the traditional Chinese medicine formula Yishen Huoxue Huatan, with a focus on its key active component sesamin.

**Methods:**

Network pharmacology was applied to predict potential targets and pathways associated with the anti-atherosclerotic activity of Yishen Huoxue Huatan. An atherosclerosis model was constructed in ApoE^−/−^ mice treated with sesamin (50 or 100 mg/kg), and endothelial injury was induced in human umbilical vein endothelial cells (HUVECs) using oxidized low-density lipoprotein (ox-LDL). Aortic pathology, lipid profiles, m^6^A RNA methylation levels, iron accumulation, oxidative stress, and ferroptosis-related markers were assessed. Ferrostatin-1 (Fer-1) was used to verify the involvement of ferroptosis in sesamin-mediated endothelial protection. The involvement of the METTL3/SREBF1 axis was further investigated using RT–qPCR, MeRIP–qPCR, mRNA stability assays, and gene-silencing approaches.

**Results:**

Network pharmacology identified sesamin as a major bioactive constituent associated with atherosclerosis-related and epigenetic regulatory pathways. In ApoE^−/−^ mice, sesamin reduced aortic lipid deposition, alleviated vascular injury, and improved dyslipidemia. In HUVECs, sesamin attenuated ox-LDL–induced endothelial injury, iron overload, lipid peroxidation, and oxidative stress. Fer-1 produced protective effects similar to those of sesamin, supporting the involvement of ferroptosis inhibition in sesamin-mediated endothelial protection. Mechanistically, sesamin downregulated METTL3 expression and reduced m^6^A modification of SREBF1 mRNA, thereby increasing SREBF1 mRNA stability and protein expression. SREBF1 silencing markedly weakened the anti-ferroptotic effects of sesamin, whereas concurrent METTL3 knockdown partially restored this protective phenotype.

**Conclusion:**

Sesamin upregulates SREBF1 expression by inhibiting METTL3-mediated m^6^A methylation, thereby alleviating ferroptosis-related alterations in vascular endothelial cells and ultimately delaying the progression of atherosclerosis.

## Introduction

Atherosclerosis (AS) is a chronic inflammatory vascular disease and the principal pathological basis of cardiovascular and cerebrovascular disorders. Despite advances in lipid-lowering and anti-inflammatory therapies, AS persists as one of the predominant causes of illness and death across the globe, highlighting the requirement for a deeper mechanistic understanding of its pathogenesis (Fan and Watanabe, 2022; Libby, 2021). AS development is driven by complex interactions among lipid metabolic imbalance, endothelial dysfunction, oxidative stress, and persistent inflammation (Hansson and Hermansson, 2011; Zhu et al., 2018). Among various forms of regulated cell death and stress responses implicated in AS, ferroptosis presents a particularly relevant mechanism. Unlike apoptosis or autophagy, ferroptosis directly links iron overload and lipid peroxidation to endothelial injury and plaque instability, both of which are central to AS progression. Moreover, emerging evidence demonstrates that ferroptosis occurs in endothelial cells, vascular smooth muscle cells, and macrophages within atherosclerotic lesions, suggesting a more direct contribution to plaque vulnerability and vascular dysfunction than other cell death pathways (Ma et al., 2022; Ouyang et al., 2021; Xu et al., 2024). This mechanistic specificity provides a rationale for focusing on ferroptosis in the context of therapeutic intervention. Ferroptosis, defined as an iron-dependent programmed cell death process characterized by lipid peroxidation, has increasingly been recognized as an important driver of AS progression.

Epigenetic regulation has gained increasing attention as a key modulator of vascular pathology. Among epigenetic mechanisms, N6-methyladenosine (m^6^A) RNA methylation—the most abundant internal modification of eukaryotic mRNA—controls gene expression by affecting RNA stability, translation, and turnover (Jian et al., 2020; Yang et al., 2023). Accumulating evidence indicates that m^6^A modification and its regulatory enzymes, including METTL3 and FTO, play essential roles in vascular cell function, lipid metabolism, and inflammatory signaling during AS development (Li et al., 2023). Notably, emerging studies suggest that m^6^A-dependent regulation of genes involved in iron homeostasis and oxidative stress may mechanistically link m^6^A modification to ferroptosis (Jiang et al., 2024; Li et al., 2024), providing a novel regulatory framework for AS. In this context, recent evidence indicates that SREBF1, traditionally known for its role in lipid metabolism, may exert context-dependent functions under oxidative stress, including regulation of ferroptosis-related antioxidant defenses (Cao et al., 2024; Yi et al., 2020).

Traditional Chinese medicine (TCM) provides a multi-target therapeutic paradigm for complex diseases such as AS. Yishen Huoxue Huatan formula, a classical compound prescription, has demonstrated anti-atherosclerotic efficacy, with its bioactive constituents, including sesamin and naringenin, shown to improve dyslipidemia and suppress inflammation and oxidative stress (Wang et al., 2024; Pham et al., 2023; Shilpa et al., 2023). Although preliminary evidence suggests that this formula may influence AS progression by modulating cell death pathways, its effects on ferroptosis and the underlying epigenetic mechanisms remain unclear. Network pharmacology analyses predict that its core components may target AS- and cell death-related pathways (Adetunji et al., 2023; Zhang et al., 2025), supporting a potential role in ferroptosis regulation.

In this investigation, we integrated network pharmacology with *in vivo* and *in vitro* experimentation to identify key active components of the Yishen Huoxue Huatan formula and to elucidate their potential roles in atherosclerosis. Focusing on a representative active compound, we investigated whether its vascular protective effects are linked to METTL3-mediated m^6^A RNA methylation, regulation of the downstream transcription factor SREBF1, and modulation of ferroptosis-related processes in vascular endothelial cells. By bridging epigenetic regulation with endothelial functional responses, this research endeavors to deliver mechanistic clarification regarding the involvement of m^6^A-dependent pathways in atherosclerosis and to support the therapeutic relevance of this traditional formula.

## Materials and methods

### Screening of active compounds and putative targets

Chemical constituents of the Yishen Huoxue Huatan formula were collected from the Traditional Chinese Medicine Systems Pharmacology Database (TCMSP; https://www.tcmsp-e.com/). The formula comprises twelve medicinal herbs: Rehmanniae Radix Praeparata, Corni Fructus, Lycii Fructus, Cuscutae Semen, Paeoniae Radix Rubra, Dioscoreae Rhizoma, Angelicae Sinensis Radix, Achyranthis Bidentatae Radix, Moutan Cortex, Citri Reticulatae Pericarpium, Pinelliae Rhizoma Praeparatum, and Poria.

Candidate active compounds were screened based on oral bioavailability (OB) ≥ 30% and drug-likeness (DL) ≥ 0.18, and their corresponding putative targets were obtained from the “Related Targets” module in TCMSP.

### Collection of atherosclerosis-related genes and target intersection

Atherosclerosis-related genes were collected from the GeneCards, OMIM, and Open Targets databases using the keyword “atherosclerosis.” Genes with relevance scores greater than three times the median in GeneCards and with a global score >0.3 in Open Targets were retained. The intersection between disease-associated genes and compound-related targets was identified for subsequent analyses.

### Protein–protein interaction (PPI) network and hub gene identification

Intersecting genes between disease-associated genes and predicted drug targets were submitted to the STRING database (https://string-db.org/) to construct a PPI network. High-confidence interactions (interaction score ≥0.700) were retained. The resulting network was rendered using Cytoscape, and hub genes were screened with the cytoHubba plugin. The ten highest-ranking genes according to Degree centrality were defined as core targets.

### Functional enrichment and network construction

Functional annotation and enrichment analyses were carried out for the intersecting genes derived from the PPI network through the R clusterProfiler package. Gene Ontology (GO) enrichment was conducted across the biological process (BP), cellular component (CC), and molecular function (MF) categories, together with Kyoto Encyclopedia of Genes and Genomes (KEGG) pathway analysis. Enrichment significance was evaluated by a hypergeometric test with Benjamini–Hochberg correction, and an false discovery rate (FDR) < 0.05 was considered statistically significant. Active constituents, their associated targets, and KEGG pathways showing significant enrichment were combined to establish a compound–target–pathway network, which was visualized using Cytoscape software.

### Molecular docking analysis

Molecular docking was conducted to explore putative structural compatibility between key SREBF1-associated active compounds and m^6^A regulatory proteins. Crystal structures of YTHDF1, YTHDF2, METTL3, FTO, and ALKBH5 were downloaded from the RCSB Protein Data Bank (https://www.rcsb.org/) and prepared in PyMOL by removing ligands, water molecules, and redundant conformations.

Two-dimensional structures of sesamin and naringenin were retrieved from TCMSP and subjected to blind docking using the CB-Dock2 platform (http://cao.labshare.cn/cb-dock2/). For each protein–ligand pair, five independent docking runs were performed, and the five conformations with the lowest binding affinities were selected for visualization and analysis.

### Animal model and treatment

Specific pathogen-free (SPF) male ApoE^−/−^ mice (6–8 weeks old, 20 ± 2 g) were used in this study. After 1 week of acclimatization under standard laboratory conditions, mice were randomly assigned to four groups (n = 6 per group): control group (Ctrl), fed a standard chow diet and administered normal saline by oral gavage daily; atherosclerosis model group (AS), fed a high-fat diet (21% fat and 0.15% cholesterol) and administered normal saline; Sesamin-50 group, fed a high-fat diet and received sesamin at 50 mg/kg/day by oral gavage; and Sesamin-100 group, fed a high-fat diet and received sesamin at 100 mg/kg/day. All treatments were continued for 12 weeks.

Sesamin, a purified lignan compound naturally present in sesame (Sesamum indicum L.), was purchased from Aladdin Biochemical Technology Co., Ltd. (Shanghai, China; catalog no. S171302; purity ≥98%). The composition of the high-fat diet was formulated following a protocol reported previously (Wang et al., 2025). Sesamin doses were selected based on prior studies demonstrating its efficacy and safety in cardiovascular and metabolic disease models (Majdalawieh et al., 2022). All mice were maintained under specific pathogen-free (SPF) conditions. All animal experiments were reviewed and approved by the Animal Ethics Committee of the Guangdong Provincial Medical Laboratory Animal Center and were conducted in accordance with the relevant guidelines and regulations for the care and use of laboratory animals.

### Cell culture and experimental treatments

Human umbilical vein endothelial cells (HUVECs) were purchased from the American Type Culture Collection (ATCC) and maintained in DMEM supplemented with 10% fetal bovine serum at 37 °C in a humidified atmosphere containing 5% CO_2_. To evaluate the cytotoxicity of sesamin, cells were treated with different concentrations of sesamin (5, 10, 25, 50, and 100 μM) for 24, 48, or 72 h. To determine the appropriate concentration of ox-LDL for establishing an endothelial injury model, HUVECs were exposed to ox-LDL at 25, 50, 100, or 150 μg/mL for 24 h. Based on these results and previous reports, the *in vitro* endothelial injury model associated with atherosclerosis was established by treating HUVECs with ox-LDL at 100 μg/mL for 24 h (Wang et al., 2025). For validation of the ox-LDL-induced endothelial injury model, HUVECs were assigned to the following groups: control, ox-LDL, and ox-LDL plus sesamin. In the ox-LDL group, cells were treated with ox-LDL at 100 μg/mL for 24 h. In the ox-LDL plus sesamin group, cells were pretreated with sesamin at 50 μM for 2 h before exposure to ox-LDL at 100 μg/mL for 24 h. Endothelial injury and lipid accumulation were evaluated by detecting cell viability, VCAM-1 and ICAM-1 protein expression, and intracellular lipid deposition.

Cells were also pretreated with sesamin at low (10 μM), medium (50 μM), or high (100 μM) concentrations for 2 h before ox-LDL stimulation, and 50 μM sesamin was selected for subsequent mechanistic experiments according to the cell viability results. For ferroptosis inhibition experiments, HUVECs were divided into control, ox-LDL, ox-LDL plus sesamin, ox-LDL plus Ferrostatin-1 (Fer-1), and ox-LDL plus sesamin and Fer-1 groups. Fer-1 was administered at 1 μM for 1 h before ox-LDL exposure, either alone or in combination with sesamin pretreatment. Cell viability, ferroptosis-related protein expression, and lipid ROS accumulation were then assessed to determine whether the protective effect of sesamin was associated with ferroptosis inhibition.

To elucidate the function of METTL3, cells were transfected with METTL3-specific small interfering RNA (si-METTL3) or a negative control siRNA (si-NC). After 24 h, cells were stimulated with ox-LDL (100 μg/mL) or pretreated with sesamin (50 μM) for 2 h before ox-LDL stimulation. To examine the role of SREBF1, cells were transfected with SREBF1-specific siRNA (si-SREBF1) or si-NC, and experimental groups included control, ox-LDL, sesamin pretreatment, and combined treatments with sesamin and si-SREBF1 and/or si-METTL3 for functional rescue experiments.

### Oil Red O and hematoxylin–eosin (H&E) staining

At the end of the experimental period, mice were anesthetized and euthanized, and aortic tissues were rapidly excised and rinsed with cold phosphate-buffered saline (PBS). For lipid deposition analysis in aortic tissues, aortic root tissues were embedded in OCT compound and sectioned into 8–10 μm-thick cryosections. The sections were fixed briefly in 4% paraformaldehyde, rinsed with PBS, and stained with Oil Red O working solution (C0157S, Beyotime, China) for 10 min in the dark, followed by hematoxylin counterstaining. For histopathological evaluation, separate aortic tissues were immersed in 4% paraformaldehyde at 4 °C for 24 h, followed by paraffin embedding and sectioning at 5 μm thickness. Sections were processed for standard H&E staining (C0105S, Beyotime).

For intracellular lipid accumulation analysis in HUVECs, cells were subjected to Oil Red O staining after the indicated treatments. Briefly, cells were washed with PBS and fixed with 4% paraformaldehyde at room temperature for 20 min. After fixation, cells were gently washed with PBS and incubated with Oil Red O working solution for 15–30 min. Excess staining solution was removed by repeated PBS washing until the background became clear. Images were captured under a light or inverted microscope. Lipid deposition in aortic tissues and Oil Red O-positive areas in HUVECs were quantified using ImageJ software.

### Measurement of serum lipid levels

At experiment completion, blood samples were collected from each mouse under deep anesthesia as part of a terminal procedure, and placed in anticoagulant-free tubes. After standing for 30 min at room temperature, samples were centrifuged (3,000 rpm, 10 min) at 4 °C to obtain serum, which was aliquoted and preserved at −80 °C for later analysis. Serum levels of triglycerides (TG; SP14979), total cholesterol (TC; SP14914), low-density lipoprotein cholesterol (LDL; SP14169), and high-density lipoprotein cholesterol (HDL; SP14182) were determined using commercial ELISA kits (Saipei, China) following manufacturers’ protocols. Absorbance was recorded at 450 nm using a Synergy HTX microplate reader (BioTek, United States), and lipid concentrations were derived from standard curves.

### Immunohistochemical analysis

Aortic tissues were preserved in 4% paraformaldehyde (pH 7.4) at 4 °C for 12 h, then cryoprotected through graded sucrose solutions (10%, 20%, and 30%) before OCT embedding. Cryosections (8 μm) were cut and mounted on glass slides. Sections were rinsed three times in PBS and treated overnight at 4 °C with primary antibodies targeting SREBF1 (A15586) or METTL3 (A21572) (both at 1:50 dilution; ABclonal, China). Following washing steps, sections were exposed to HRP-conjugated secondary antibodies at room temperature for 1 h, followed by visualization with DAB substrate (P0201M, Beyotime). Sections were counterstained with hematoxylin, mounted with neutral resin, and examined under an Olympus BX53 microscope. Protein expression was semi-quantitatively analyzed using ImageJ software.

### Measurement of iron levels

Aortic tissues from mice or HUVECs were collected and rinsed in ice-cold saline to remove residual blood. Approximately 50 mg of tissue or harvested cells were processed according to the manufacturer’s instructions using an Iron Assay Kit (S1066S, Beyotime). Briefly, samples were homogenized and incubated with the reaction solution for 30 min at 37 °C, followed by measurement of absorbance at the specified wavelength using a Synergy HTX microplate reader (BioTek). Ferrous iron (Fe^2+^) levels were derived from standard curves and adjusted for protein content (expressed as nmol/mg protein or μmol/g protein).

### Immunoblotting analysis

Aortic samples or HUVECs were extracted in chilled RIPA buffer (P0013B, Beyotime) containing protease/phosphatase inhibitor cocktails. Lysates were homogenized on ice, incubated at 4 °C for 30 min, and centrifuged at 12,000 rpm for 15 min. Total protein concentrations were determined using a BCA protein assay kit (P0009, Beyotime). Equal amounts of protein samples were mixed with loading buffer, denatured, separated by SDS–PAGE, and transferred onto PVDF membranes.

After blocking with 5% nonfat milk in TBST at room temperature, the membranes were incubated overnight at 4 °C with primary antibodies against ACSL4 (A20414), GPX4 (A13309), xCT (A13685), SREBF1 (A15586), METTL3 (A21572), and β-actin (AC038) (all 1:1,000, except β-actin 1:5,000; ABclonal, China). For the supplementary endothelial injury and ferroptosis inhibition experiments, membranes were additionally incubated with primary antibodies against VCAM-1 (ab134047, 1:2000, Abcam), ICAM-1 (ab282575, 1:1,000, Abcam), GPX4 (ab125066, 1:10,000, Abcam), ACSL4 (ab155282, 1:10,000, Abcam), and β-actin (81115-1-RR, 1:10,000, Proteintech). After washing with TBST, the membranes were incubated with HRP-conjugated secondary antibodies for 1 h at room temperature.

Protein signals were visualized using enhanced chemiluminescence reagents and captured with a chemiluminescence imaging system. Band intensities were quantified using ImageJ software and normalized to β-actin.

### Quantification of global m^6^A RNA methylation

Total RNA was isolated from aortic tissues or HUVECs with TRIzol reagent. Global m^6^A RNA methylation levels were quantified by a commercial m^6^A RNA Methylation Quantification Kit (EpiQuik, EpiGentek, United States) according to the manufacturer’s instructions. Briefly, RNA samples were incubated with capture antibodies in 96-well plates at 37 °C for 1 h, followed by incubation with detection antibodies. After substrate development and reaction termination, absorbance was measured at 450 nm using a Synergy HTX microplate reader (BioTek). Total m^6^A levels were calculated from standard curves and expressed as relative abundance or percentage.

### Cell viability assay

HUVECs were seeded into 96-well plates at a density of 5 × 10^4^ cells/mL, with 100 μL of cell suspension added to each well, and cultured for 24 h at 37 °C in a 5% CO_2_ incubator. After the indicated treatments with sesamin, ox-LDL, and/or Fer-1, cell viability was assessed using a Cell Counting Kit-8 (CCK-8; C0037, Beyotime). Briefly, 10 μL of CCK-8 reagent was added to each well, followed by incubation at 37 °C for 2 h in the dark. Absorbance was measured at 450 nm using a microplate reader, and cell viability was calculated relative to the control group.

### Measurement of oxidative stress–related parameters

HUVECs or tissue homogenates were subjected to oxidative stress–related assays. Lipid reactive oxygen species (ROS) levels were quantified with the C11-BODIPY 581/591 fluorescent probe (S1111M, Beyotime). Cells were treated with C11-BODIPY (10 μM) at 37 °C for 30 min in darkness, followed by PBS washing. Fluorescence intensity was detected with a fluorescence microplate reader (Synergy™ 2 SL, BioTek) with excitation/emission at 581/591 nm. Lipid ROS levels were calculated based on fluorescence intensity. For imaging-based detection of lipid ROS in HUVECs, cells were incubated with C11-BODIPY 581/591 working solution at 37 °C in the dark for 10–30 min. After staining, cells were washed twice with PBS and observed under a fluorescence microscope. The oxidized/reduced fluorescence ratio was quantified using ImageJ software.

Malondialdehyde (MDA; S0131S), superoxide dismutase (SOD; S0101M), and the reduced/oxidized glutathione (GSH/GSSG) ratio (S0053) were analyzed using commercial kits (Beyotime). For MDA determination, samples were mixed with thiobarbituric acid (TBA) reagent, heated at 95 °C for 40 min, cooled, centrifuged, and absorbance was recorded at 532 nm. SOD activity was assessed using the WST-8 method at 450 nm. The GSH/GSSG ratio was evaluated *via* reaction with DTNB and measured at 412 nm. All values were expressed after normalization to protein concentration (nmol/mg or U/mg protein).

### RT-qPCR analysis

Total RNA extraction from HUVECs or tissues was performed using TRIzol reagent. RNA yield and purity were evaluated using a NanoDrop spectrophotometer. One microgram of RNA was subjected to reverse transcription using HiScript III All-in-One RT SuperMix for qPCR (R333-01, Vazyme, China). Quantitative real-time PCR was performed using ChamQ SYBR Color qPCR Master Mix (Q411-02, Vazyme) on a LightCycler 480 system (Roche, United States). The amplification conditions were as follows: initial denaturation at 95 °C for 5 min, followed by 40 cycles of 95 °C for 10 s and 60 °C for 30 s. GAPDH was used as an internal reference gene, and relative gene expression levels were calculated using the 2^−ΔΔCt^ method. Primer sequences are listed in Table 1.

**TABLE 1:** Primer sequences.

Gene	Primers	Primer sequences (5′-3′)
*SREBF1*	F	GGC​GGA​ACC​ATC​TTG​GCA​A
	R	TCT​CCT​GCT​TGA​GTT​TCT​GG
*METTL3*	F	ACA​GAT​GAT​GAG​ATG​CGC​AG
	R	TCA​TCT​GGT​TTA​TGA​CTG​GTG​G
*METTL14*	F	TGG​ACC​AAC​GCT​TAC​AAA​TAG​C
	R	CTC​TTT​CTC​CTC​GGA​AGT​TAG
*FTO*	F	ATG​AGG​TCG​AGT​TTG​AGT​GG
	R	CTG​AGT​TCT​GAA​ACG​ATG​TCT​G
*YTHDF1*	F	GTA​CTC​CAT​CTG​GTG​TAG​CA
	R	GAC​ATC​AAA​CTT​CCC​CTT​CC
*GAPDH*	F	AAG​ATC​ATC​AGC​AAT​GCC​TCC
	R	AGG​TTT​TTC​TAG​ACG​GCA​GG

### MeRIP–qPCR analysis

Total RNA obtained from HUVECs was subjected to m^6^A RNA immunoprecipitation (MeRIP) using a commercial MeRIP kit (C11051-1, RiboBio, China). Briefly, fragmented RNA was treated overnight at 4 °C with an anti-m^6^A antibody, followed by binding to Protein A/G magnetic beads to capture m^6^A-modified RNA. After washing, recovered RNA was purified and subjected to qPCR. Five percent of total RNA was utilized as the input control, and IgG was used as a negative control. m^6^A enrichment on SREBF1 mRNA was expressed as the IP/Input ratio. qPCR conditions were identical to those described above.

### Statistical analysis

All results are expressed as the mean ± standard deviation (SD). Statistical evaluation was carried out using GraphPad Prism 9.0 software. Data normality and homogeneity of variance were assessed using the Shapiro–Wilk and Levene tests, respectively. For data meeting parametric assumptions, comparisons among multiple groups were performed using one-way analysis of variance (ANOVA), followed by Tukey’s *post hoc* test. Repeated-measures data were analyzed using repeated-measures ANOVA with Bonferroni correction. A P value <0.05 was considered statistically significant.

## Results

### Integrated network pharmacology analysis identifies potential anti-atherosclerotic targets and pathways of Yishen Huoxue Huatan formula

**FIGURE 1:**
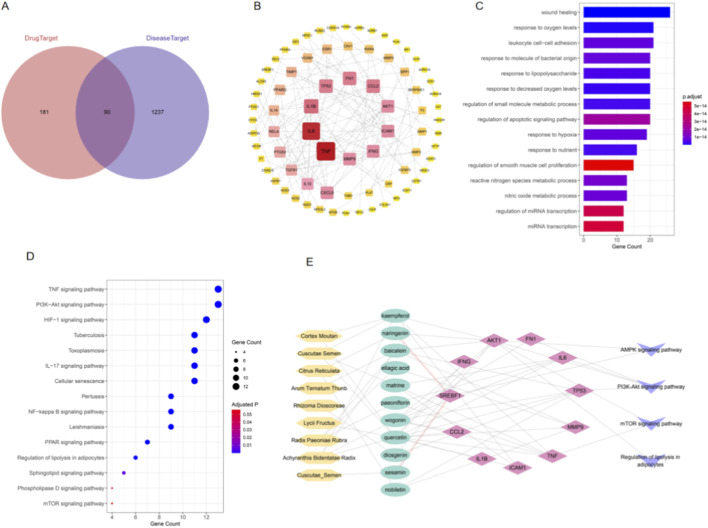
Integrated network pharmacology analysis of Yishen Huoxue Huatan formula in atherosclerosis. **(A)** Venn diagram showing the overlap between predicted targets of active compounds in the formula and atherosclerosis-related genes. **(B)** Protein–protein interaction (PPI) network of the intersecting targets. **(C)** GO enrichment analysis of intersecting genes. Representative terms related to inflammatory response, oxidative stress, metabolic regulation, and RNA/epigenetic regulation are shown. **(D)** KEGG pathway enrichment analysis of intersecting targets. **(E)** Compound–target–pathway network showing the multi-component, multi-target, and multi-pathway characteristics of the formula.

To explore the potential molecular basis underlying the anti-atherosclerotic effects of Yishen Huoxue Huatan formula, network pharmacology analysis was first performed. Active compounds and their putative targets were screened from the TCMSP database, and atherosclerosis-related genes were collected from GeneCards, OMIM, and Open Targets databases. After intersection analysis, 90 overlapping targets were identified between formula-related targets and atherosclerosis-associated genes, suggesting that these genes may represent the potential therapeutic targets of Yishen Huoxue Huatan formula in atherosclerosis (Figure 1A).

To further clarify the interaction relationships among these intersecting genes, a PPI network was constructed using the STRING database. Several inflammation- and vascular injury-related genes, including TNF, IL6, IL1B, TP53, AKT1, ICAM1, CCL2, MMP9, FN1, and IFNG, occupied central positions in the network, indicating that inflammatory regulation, endothelial activation, and vascular remodeling may be important biological processes involved in the formula-mediated anti-atherosclerotic effects (Figure 1B).

GO enrichment analysis showed that the intersecting targets were significantly enriched in biological processes closely related to atherosclerosis progression, including wound healing, response to oxygen levels, leukocyte cell–cell adhesion, response to lipopolysaccharide, regulation of apoptotic signaling pathway, response to hypoxia, regulation of smooth muscle cell proliferation, reactive nitrogen species metabolic process, and nitric oxide metabolic process (Figure 1C). Notably, enrichment terms associated with regulation of miRNA transcription and miRNA transcription were identified, indicating the involvement of RNA-level regulatory mechanisms. These terms, combined with prior knowledge that METTL3, FTO, and YTHDF proteins modulate endothelial function and ferroptosis, provided a rationale for focusing on m6A modification in subsequent experiments.

KEGG pathway analysis further revealed that these intersecting targets were mainly enriched in inflammation-related, metabolic, and stress-response pathways, including TNF signaling pathway, PI3K–Akt signaling pathway, HIF-1 signaling pathway, IL-17 signaling pathway, NF-κB signaling pathway, PPAR signaling pathway, and mTOR signaling pathway (Figure 1D). These pathways are closely associated with endothelial dysfunction, oxidative stress, lipid metabolism, inflammatory activation, and cell survival regulation during atherosclerosis.

In addition to these dominant inflammatory, metabolic, and stress-response pathways, KEGG enrichment analysis also identified significant enrichment of cellular senescence and the microRNA-related pathway (Supplementary Table S1). Cellular senescence involved 11 intersecting genes and showed strong enrichment, suggesting that the formula-associated targets may participate in stress-induced cell fate regulation. The enrichment of the microRNA-related pathway further indicated the potential involvement of RNA-associated post-transcriptional regulatory networks.

To further visualize the multi-component and multi-target characteristics of Yishen Huoxue Huatan formula, a compound–target–pathway network was constructed. The network showed that multiple active compounds, including sesamin, naringenin, kaempferol, baicalein, wogonin, paeoniflorin, and diosgenin, were connected with key targets and enriched signaling pathways, reflecting the multi-component, multi-target, and multi-pathway pharmacological features of the formula (Figure 1E). Among these compounds, sesamin was linked to several atherosclerosis-related targets and pathways and has been reported to possess lipid-lowering, antioxidant, anti-inflammatory, and vascular protective effects. In addition, the enrichment of RNA regulatory terms and the involvement of stress- and metabolism-related pathways provided a rationale for further investigating whether sesamin exerts endothelial protective effects through RNA modification-related mechanisms. Therefore, sesamin was selected as a representative active compound for subsequent experimental validation, with particular focus on the potential involvement of the m^6^A-related METTL3/SREBF1 axis.

### Molecular docking analysis

To explore the potential structural compatibility between representative active compounds and m^6^A-related regulatory proteins, molecular docking analyses were performed. Sesamin and naringenin were docked against YTHDF1, YTHDF2, METTL3, FTO, and ALKBH5. For each protein–ligand pair, multiple docking conformations were generated, and the lowest-energy conformations were selected for comparative analysis. The docking results showed that sesamin exhibited relatively lower predicted binding energies than naringenin across the analyzed m^6^A-related proteins. Representative binding poses suggested that sesamin could adopt favorable conformations within the predicted binding regions of YTHDF1, YTHDF2, FTO, and ALKBH5 through noncovalent interactions, including hydrogen bonding and hydrophobic contacts (Supplementary Figure S1). These *in silico* observations provide preliminary structural support for a potential association between sesamin and m^6^A regulatory proteins, without implying direct functional inhibition.

### Sesamin attenuates lipid deposition and pathological changes in atherosclerotic mice

**FIGURE 2:**
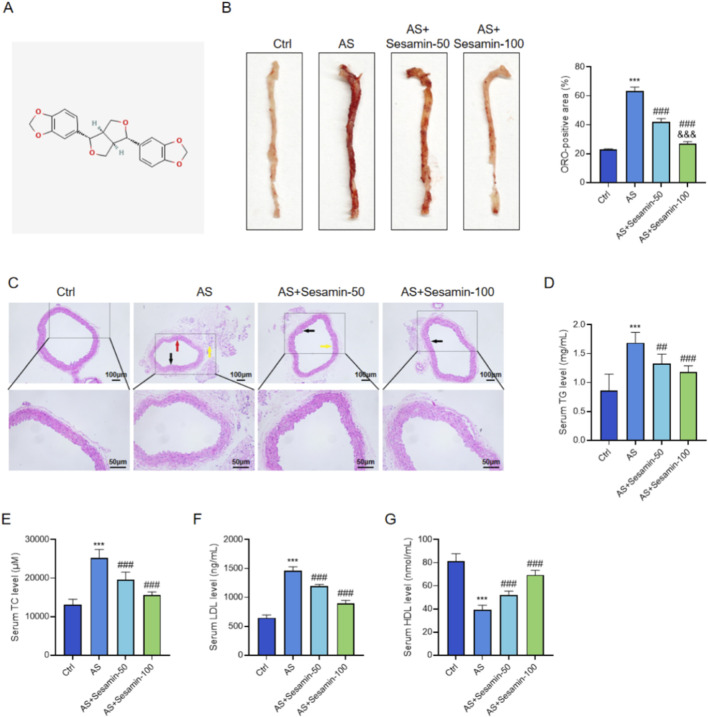
Sesamin attenuates lipid deposition and pathological changes in atherosclerotic mice. **(A)** Chemical structure of sesamin. **(B)** Representative Oil Red O staining of aortas showing lipid accumulation in each group. **(C)** Representative H&E staining of aortic cross-sections illustrating histopathological changes. **(D–G)** Serum concentrations of TG, TC, LDL, and HDL measured by ELISA. Data are presented as mean ± SD (n = 6 mice per group). ***p < 0.001 vs. Ctrl group; ##p < 0.01, ###p < 0.001 vs. AS group; &&&p < 0.001 vs. AS + Sesamin-50 group.

To investigate the anti-atherosclerotic effects of sesamin, an ApoE^−/−^ mouse model of atherosclerosis was established. The chemical structure of sesamin used in this study is shown in Figure 2A. Oil Red O staining showed extensive lipid deposition along the aortic wall in AS model mice relative to control animals, confirming successful model establishment. In contrast, sesamin treatment markedly reduced aortic lipid accumulation, with a dose-dependent effect favoring the Sesamin-100 mg/kg group (Figure 2B).

H&E staining further revealed histopathological alterations in the aortic tissue. The Ctrl group exhibited intact vascular architecture with a smooth intima, whereas the AS group showed marked thickening of the vascular wall, prominent foam cell accumulation in the subintimal region, and formation of typical atherosclerotic plaques with disorganized structure. Sesamin administration notably alleviated these pathological changes, as reflected by diminished vascular wall thickening, decreased plaque area, and improved tissue morphology (Figure 2C).

Serum lipid profiles were also assessed (Figures 2D–G). Relative to the Ctrl group, AS mice displayed markedly elevated levels of TC, TG, and LDL, accompanied by a marked reduction in HDL. Sesamin administration, particularly at 100 mg/kg, effectively reversed these alterations by lowering TC, TG, and LDL levels and elevating HDL levels. Collectively, these results demonstrate that sesamin reduces aortic lipid deposition, alleviates vascular pathological injury, and improves dyslipidemia in ApoE^−/−^ mice.

### Sesamin upregulates SREBF1 *via* m^6^A regulation and alleviates ferroptosis in aortic tissue

**FIGURE 3:**
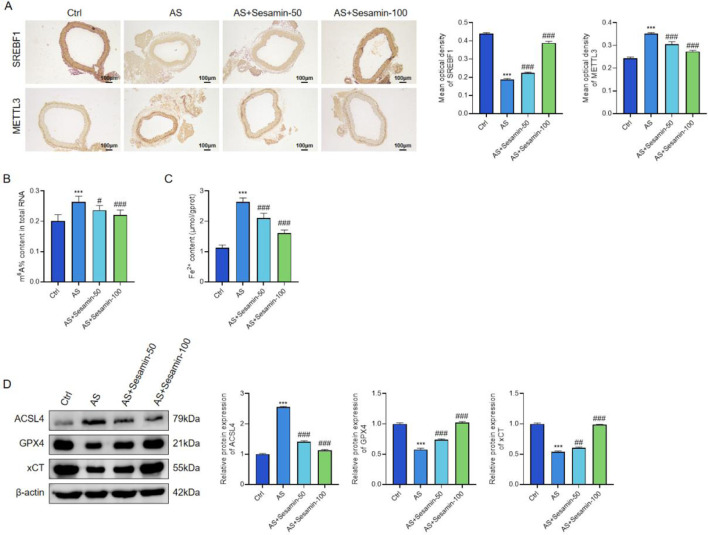
Sesamin modulates m^6^A methylation and ferroptosis-related markers in aortic tissue. **(A)** Representative immunohistochemical staining of SREBF1 and METTL3 protein expression in aortic sections from each group. **(B)** Quantification of global m^6^A RNA methylation levels in aortic tissues. **(C)** Fe^2+^ content in aortic tissues determined using an iron assay kit. **(D)** Immunoblotting analysis of ACSL4, GPX4, and xCT protein levels in aortic tissues. Results are shown as mean ± SD (n = 6 mice per group). ***p < 0.001 vs. Ctrl group; #p < 0.05, ##p < 0.01, ###p < 0.001 vs. AS group.

To further elucidate the mechanisms underlying the anti-atherosclerotic effects of sesamin, key indicators associated with ferroptosis were examined. Immunohistochemical analysis revealed that SREBF1 protein expression was significantly reduced, whereas METTL3 expression was markedly increased in aortic tissues of AS mice compared with controls. Sesamin treatment reversed these changes, leading to increased SREBF1 expression and decreased METTL3 expression, with more pronounced effects observed at the higher dose (Figure 3A). Consistently, global m^6^A RNA methylation levels were markedly upregulated in aortic tissues of AS mice, whereas sesamin treatment substantially reduced this abnormal increase, showing a stronger effect at the higher dose (Figure 3B). Given the close association between ferroptosis and iron metabolism, Fe^2+^ levels were measured and were notably elevated in the AS group. Sesamin treatment markedly reduced Fe^2+^ accumulation in aortic tissues (Figure 3C), indicating attenuation of AS-associated iron overload. Western blotting further revealed a marked upregulation of the pro-ferroptotic protein ACSL4, accompanied by downregulation of the anti-ferroptotic proteins GPX4 and xCT in the AS group. These aberrant expression patterns were substantially reversed by sesamin treatment, particularly at the higher dose (Figure 3D). Collectively, these observations show that sesamin is linked to reduced m^6^A methylation, increased SREBF1 expression, and attenuation of ferroptosis-related alterations in atherosclerotic aortic tissue.

### Validation of the ox-LDL-induced endothelial injury model in HUVECs

To verify the establishment of the ox-LDL-induced endothelial injury model, HUVECs were treated with different concentrations of ox-LDL for 24 h. CCK-8 assays showed that ox-LDL reduced cell viability in a concentration-dependent manner, with 100 μg/mL ox-LDL inducing a significant but moderate decrease in cell viability, while 150 μg/mL caused more pronounced cytotoxicity. Therefore, 100 μg/mL ox-LDL was selected for subsequent experiments (Supplementary Figure S2A). Western blot analysis further showed that ox-LDL stimulation markedly increased the protein expression levels of the endothelial adhesion molecules VCAM-1 and ICAM-1, whereas sesamin pretreatment significantly reduced their expression (Supplementary Figure S2B). Consistently, Oil Red O staining revealed obvious intracellular lipid accumulation after ox-LDL exposure, which was markedly attenuated by sesamin treatment (Supplementary Figure S2C). These results confirmed that ox-LDL successfully induced endothelial injury and lipid deposition in HUVECs, and that sesamin exerted a protective effect against ox-LDL-induced endothelial dysfunction.

### Sesamin attenuates ferroptosis-related features and improves oxidative stress in vitro

**FIGURE 4:**
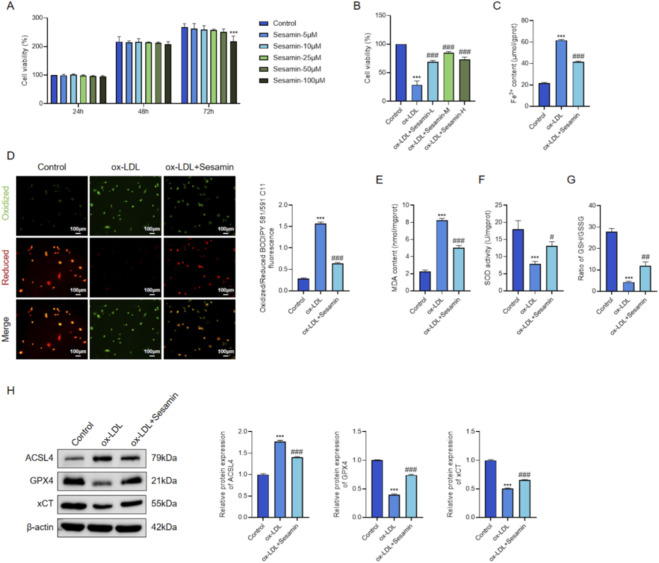
Sesamin attenuates ox-LDL–induced ferroptosis-related alterations and oxidative stress *in vitro*. **(A)** Cell viability of endothelial cells receiving graded doses of sesamin (5, 10, 25, 50, and 100 μM) assessed by the CCK-8 assay. **(B)** Cell viability of control, ox-LDL–treated, and ox-LDL + sesamin–treated cells (10, 50, and 100 μM) determined by the CCK-8 assay. **(C)** Intracellular Fe^2+^ levels in control, ox-LDL, and ox-LDL + sesamin (50 μM) groups measured using an iron assay kit. **(D)** Representative images of intracellular lipid ROS detected by the C11-BODIPY 581/591 probe in each group. **(E–G)** Levels of MDA, SOD activity, and the GSH/GSSG ratio in control, ox-LDL, and ox-LDL + sesamin–treated cells. **(H)** Western blot analysis of ferroptosis-related proteins ACSL4, GPX4, and xCT in endothelial cells. Data are presented as mean ± SD (n = 3 independent experiments). ***p < 0.001 vs. control group; #p < 0.05, ##p < 0.01, ###p < 0.001 vs. ox-LDL group.

To validate the direct cytoprotective role of sesamin against endothelial injury at the cellular level, its impact on cell viability was first evaluated. CCK-8 assays indicated that sesamin at concentrations of 5–50 μM did not significantly affect the viability of HUVECs after 24, 48, or 72 h of treatment. However, exposure to 100 μM sesamin for 72 h notably reduced cell viability, indicating cytotoxicity at high concentrations (Figure 4A). Accordingly, 50 μM sesamin was selected for subsequent experiments. In an ox-LDL-triggered endothelial injury model, exposure to ox-LDL (100 μg/mL, 24 h) significantly decreased HUVEC viability compared with control cells. Sesamin pretreatment (10, 50, or 100 μM) markedly restored cell viability, with the greatest improvement observed at 50 μM. The slightly reduced efficacy at 100 μM may be attributable to its potential inhibitory effects at higher concentrations (Figure 4B).

Mechanistically, ox-LDL stimulation markedly increased intracellular Fe^2+^ levels, which were significantly attenuated by co-treatment with 50 μM sesamin (Figure 4C). Lipid peroxidation assessed using the C11-BODIPY 581/591 probe showed a dramatic elevation in lipid ROS following ox-LDL exposure, whereas sesamin treatment effectively suppressed ox-LDL-induced lipid peroxidation (Figure 4D). In parallel, ox-LDL treatment significantly increased malondialdehyde (MDA) levels and reduced superoxide dismutase (SOD) activity and the GSH/GSSG ratio, indicating severe oxidative stress. These alterations were markedly reversed by sesamin treatment (Figures 4E–G).

Consistent with these findings, Western blot analysis showed that ox-LDL significantly upregulated ACSL4 expression and downregulated GPX4 and xCT expression, whereas sesamin treatment restored their expression toward basal levels (Figure 4H).

Collectively, these results indicate that sesamin, at non-cytotoxic concentrations, alleviates ox-LDL-induced endothelial injury, accompanied by reduced iron accumulation, decreased lipid peroxidation, and improved oxidative stress–related parameters.

### Sesamin-mediated endothelial protection is associated with ferroptosis inhibition

To further determine whether the protective effect of sesamin was associated with ferroptosis inhibition, HUVECs were treated with ox-LDL in the presence of sesamin and/or the ferroptosis inhibitor Ferrostatin-1 (Fer-1). CCK-8 assays showed that ox-LDL significantly reduced cell viability, whereas sesamin or Fer-1 treatment markedly restored cell viability. Notably, combined treatment with sesamin and Fer-1 did not produce an obvious additional increase compared with either treatment alone (Supplementary Figure S3A). Western blot analysis showed that ox-LDL decreased GPX4 expression and increased ACSL4 expression, while sesamin and Fer-1 similarly reversed these ferroptosis-related protein changes (Supplementary Figure S3B). Consistently, C11-BODIPY staining demonstrated that ox-LDL markedly increased lipid ROS accumulation, as indicated by enhanced oxidized fluorescence and an increased oxidized/reduced fluorescence ratio. These effects were significantly attenuated by sesamin, Fer-1, or their combined treatment (Supplementary Figure S3C). Together, these findings further support that sesamin alleviates ox-LDL-induced endothelial injury, at least partly, by suppressing ferroptosis-related lipid peroxidation.

### Sesamin upregulates SREBF1 expression by regulating m^6^A RNA methylation

**FIGURE 5:**
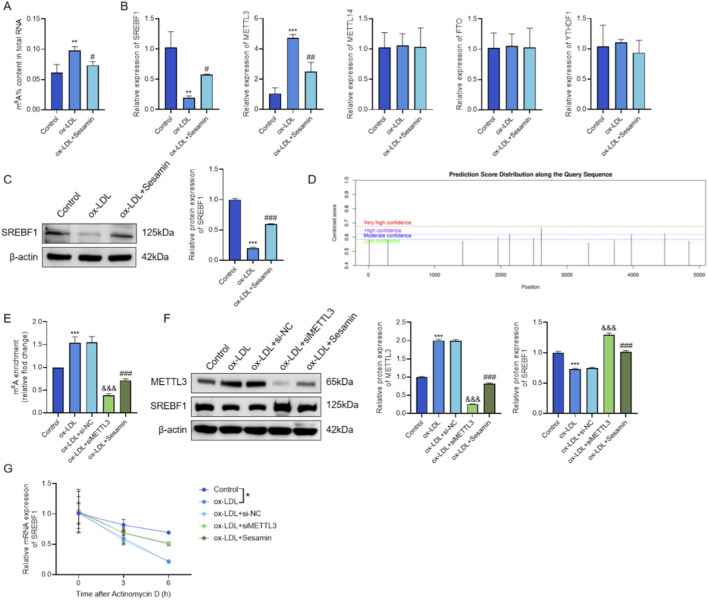
Sesamin regulates SREBF1 expression through m^6^A RNA methylation in endothelial cells. **(A)** Quantification of global m^6^A RNA methylation levels in control, ox-LDL, and ox-LDL + sesamin groups. **(B)** RT–qPCR analysis of SREBF1, METTL3, METTL14, FTO, and YTHDF1 mRNA expression in each group. **(C)** Western blot analysis of SREBF1 protein expression in control, ox-LDL, and ox-LDL + sesamin–treated cells. **(D)** Prediction of potential m^6^A modification sites on SREBF1 mRNA using the SRAMP online tool. **(E)** MeRIP–qPCR analysis of m^6^A enrichment on SREBF1 mRNA in control, ox-LDL, ox-LDL + si-NC, ox-LDL + si-METTL3, and ox-LDL + sesamin groups. **(F)** Western blot analysis of METTL3 and SREBF1 protein expression in the indicated groups. **(G)** Actinomycin D chase assay evaluating SREBF1 mRNA stability in the indicated groups. Data are expressed as mean ± SD (n = 3 independent experiments). *p < 0.05, **p < 0.01, ***p < 0.001 vs. control group; #p < 0.05, ##p < 0.01, ###p < 0.001 vs. ox-LDL group; &&&p < 0.001 vs. ox-LDL + si-NC group.

To clarify the molecular basis of the protective actions of sesamin, we examined the involvement of m^6^A RNA methylation in this process. Quantification of global m^6^A levels revealed that ox-LDL exposure markedly elevated overall m^6^A modification in endothelial cells, whereas co-treatment with sesamin (50 μM) substantially reversed this increase (Figure 5A). RT–qPCR analysis of m^6^A-related regulatory factors and downstream target genes showed that ox-LDL exposure significantly reduced SREBF1 mRNA expression while upregulating the core m^6^A methyltransferase component METTL3. Sesamin treatment effectively counteracted these changes by upregulating SREBF1 and downregulating METTL3 expression. At the protein level, Western blot analysis showed a consistent trend: ox-LDL markedly suppressed SREBF1 protein expression, which was restored to near-baseline levels following sesamin treatment (Figures 5B,C). These findings indicate that SREBF1 expression may be modulated *via* m^6^A modification.

To strengthen support for this hypothesis, m^6^A site prediction using the SRAMP online database identified multiple potential m^6^A modification sites within the coding sequence (CDS) and 3′ untranslated region (3′UTR) of SREBF1 mRNA (Figure 5D). We next performed MeRIP–qPCR to directly assess m^6^A enrichment on SREBF1 mRNA. As compared to the control group, ox-LDL exposure markedly elevated m^6^A enrichment on SREBF1 transcripts (Figure 5E). To validate the involvement of METTL3, siRNA-mediated knockdown of METTL3 or treatment with sesamin markedly reduced ox-LDL–induced m^6^A hyperenrichment on SREBF1 mRNA (Supplementary Figure S4A–B). Correspondingly, Western blot analysis demonstrated that either METTL3 knockdown or sesamin treatment reversed ox-LDL–induced METTL3 upregulation and SREBF1 downregulation at the protein level (Figure 5F).

To determine the functional consequence of m^6^A modification on SREBF1 mRNA, actinomycin D (ActD) chase assays were performed to evaluate mRNA stability. Ox-LDL treatment significantly reduced the stability of SREBF1 mRNA, whereas METTL3 knockdown or sesamin treatment markedly enhanced SREBF1 mRNA stability and delayed its degradation (Figure 5G). Collectively, these results demonstrate that ox-LDL is associated with increased METTL3 expression, enhanced m^6^A modification of SREBF1 mRNA, and reduced SREBF1 mRNA stability and expression. In contrast, sesamin treatment attenuated these ox-LDL–induced alterations by reducing METTL3 expression, decreasing m^6^A enrichment on SREBF1 mRNA, and improving its stability.

### Sesamin regulates ferroptosis-related alterations and oxidative stress *via* the m^6^A–SREBF1 axis

**FIGURE 6:**
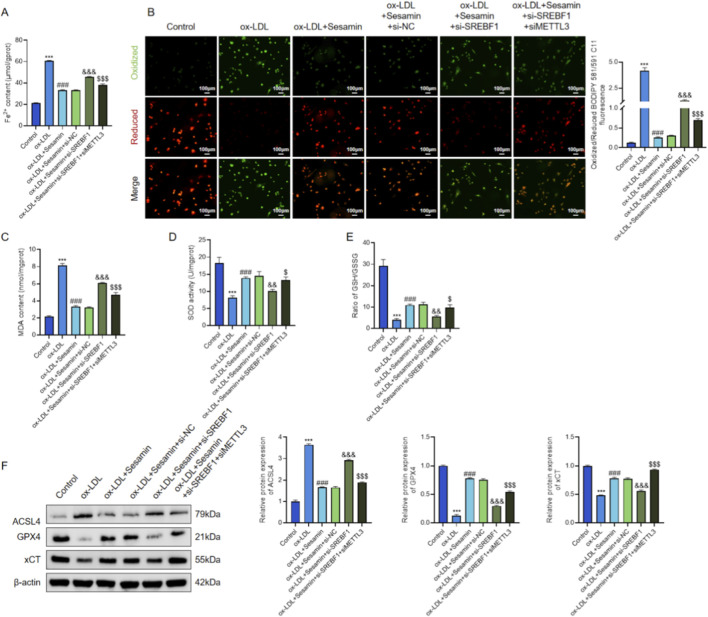
Functional validation of the METTL3–SREBF1 pathway in sesamin-mediated suppression of endothelial ferroptosis. **(A)** Intracellular Fe^2+^ levels in control, ox-LDL–treated, ox-LDL + sesamin–treated, ox-LDL + sesamin + si-NC, ox-LDL + sesamin + si-SREBF1, and ox-LDL + sesamin + si-SREBF1 + si-METTL3 groups. **(B)** Representative images and quantitative analysis of intracellular lipid ROS detected by the C11-BODIPY 581/591 probe in the indicated groups. **(C–E)** Levels of MDA, SOD activity, and the GSH/GSSG ratio in the indicated groups. **(F)** Western blot analysis of ferroptosis-related proteins ACSL4, GPX4, and xCT in the indicated groups. Data are presented as mean ± SD (n = 3 independent experiments). ***p < 0.001 vs. control group; ###p < 0.001 vs. ox-LDL group; &&p < 0.01, &&&p < 0.001 vs. ox-LDL + sesamin + si-NC group; $p < 0.05, $$p < 0.01, $$$p < 0.001 vs. ox-LDL + sesamin + si-SREBF1 group.

To clarify the role of SREBF1 in sesamin-mediated protection, double-knockdown experiments were performed. Intracellular Fe^2+^ levels showed that SREBF1 silencing weakened the ability of sesamin to reduce ox-LDL–induced iron accumulation, whereas concurrent METTL3 knockdown partially mitigated the iron overload observed under SREBF1 deficiency (Figure 6A). Consistently, C11-BODIPY staining indicated that SREBF1 knockdown attenuated the protective effect of sesamin on lipid peroxidation, while co-silencing METTL3 partially reduced lipid ROS levels (Figure 6B). Oxidative stress–related parameters (MDA, SOD activity, and GSH/GSSG ratio) exhibited similar trends (Figures 6C–E). Western blotting further showed that SREBF1 silencing blunted sesamin-associated changes in ACSL4, GPX4, and xCT, whereas METTL3 co-silencing partially restored these protein alterations (Figure 6F). Collectively, these results indicate that SREBF1 is an important downstream mediator of sesamin-associated protection against ferroptosis-related alterations in endothelial cells and support the involvement of the METTL3–m^6^A–SREBF1 axis in regulating iron accumulation, lipid peroxidation, and ferroptosis-related marker expression under ox-LDL stress.

## Discussion

In this study, by integrating network pharmacology–based predictions with experimental validation, we systematically elucidated a previously unrecognized mechanism by which sesamin, a key active component of the traditional Chinese medicine formula Yishen Huoxue Huatan, exerts anti-atherosclerotic effects. Specifically, sesamin was shown to modulate METTL3-mediated m^6^A RNA methylation, leading to upregulation of the transcription factor SREBF1, thereby alleviating ferroptosis-related alterations in vascular endothelial cells and ultimately attenuating atherosclerosis progression. These results offer a scientific rationale for the clinical translation of Yishen Huoxue Huatan and offer experimental evidence for exploring the pathogenesis and therapeutic intervention of atherosclerosis from the emerging perspective of the m^6^A methylation–ferroptosis axis.

Our network pharmacology analysis suggested that Yishen Huoxue Huatan formula and its active constituents are markedly enriched in pathways associated with lipid metabolism, inflammation, and cell death, with potential associations with m^6^A regulatory factors. This provided a directional framework for subsequent mechanistic investigations. Sesamin, a lignan compound derived from sesame oil, has been widely shown to exert multiple pharmacological effects, such as antioxidant, anti-inflammatory, lipid-modulatory, and endothelial-protective properties, and has shown promising therapeutic potential in cardiovascular diseases (Dalibalta et al., 2020; Ghaderi et al., 2023; Majdalawieh et al., 2020). Prior investigations have directly demonstrated the ability of sesamin to mitigate atherosclerosis in animal models (Hadipour et al., 2023). For example, Wu et al. reported that sesamin reduced aortic atherosclerotic lesion area in ApoE-deficient mice by suppressing ICAM-1 expression (Wu et al., 2010). Nakamura et al. further showed that sesamin alleviated hepatic inflammation induced by a diet enriched in fat and cholesterol and decreased plasma platelet-activating factor acetylhydrolase (PAF-AH) activity, thereby decreasing LDL susceptibility to oxidation (Nakamura et al., 2020).

These studies laid a solid foundation for the present work; however, the precise molecular mechanisms of sesamin, particularly those involving epigenetic regulation and novel forms of regulated cell death, remained largely unexplored. Although previous reports have suggested that sesamin can inhibit ferroptosis by downregulating HMOX1, FoxO1, or the Nrf2/SLC7A11/GPX4 pathway (Ren et al., 2025; Ren et al., 2021; Yang et al., 2025; Zhu et al., 2025), whether sesamin exerts its anti-atherosclerotic effects through modulation of ferroptosis and whether this process is linked to m^6^A RNA methylation had not been previously reported. Our subsequent *in vivo* and *in vitro* experiments demonstrated that sesamin significantly attenuates aortic lipid deposition and pathological injury in ApoE^−/−^ mice while improving lipid profiles, confirming its potent anti-atherosclerotic efficacy. More importantly, this research provides, to our knowledge, the first mechanistic support linking sesamin-mediated m^6^A dysregulation to ferroptosis-related endothelial injury during AS progression. In both animal and cellular models, sesamin reversed ox-LDL–induced increases in global m^6^A levels and ameliorated key features of ferroptosis, including iron accumulation, lipid peroxidation, and impairment of the antioxidant defense system (GPX4/xCT). By linking sesamin’s protective effects to epigenetic m^6^A regulation and ferroptosis, our study substantially expands the current understanding of its pharmacological mechanisms.

The principal finding of this work is that the METTL3/SREBF1 axis functions as a critical signaling pathway underlying the protective actions of sesamin. Through multilayered experimental evidence, we demonstrate that sesamin downregulates the m^6^A methyltransferase METTL3, thereby selectively reducing m^6^A modification on SREBF1 mRNA. This finding is well supported by existing literature. Accumulating evidence indicates that METTL3 functions as a pathogenic “accelerator” in atherosclerosis progression (Chen et al., 2023; Dong et al., 2025). In vascular endothelial cells, METTL3-dependent m^6^A modification has been reported to exacerbate endothelial dysfunction and inflammatory responses by stabilizing pro-inflammatory mRNAs or promoting pro-apoptotic signaling, ultimately accelerating AS development (Ping et al., 2025; Zhao et al., 2025). Our findings are in strong agreement with these results and further extend the pathological role of METTL3 to the regulation of ferroptosis, a newly recognized type of regulated cell death.

Importantly, using MeRIP–qPCR and actinomycin D chase assays, we demonstrate that ox-LDL promotes METTL3-dependent m^6^A modification on SREBF1 mRNA, thereby accelerating its degradation and reducing SREBF1 protein expression. In contrast, sesamin suppresses METTL3 expression, enhances SREBF1 mRNA stability, and restores its expression. The downstream effector SREBF1 exhibits a more complex and context-dependent role. Traditionally, SREBF1 has been regarded as a master regulator of lipogenesis, promoting fatty acid and cholesterol synthesis and often considered detrimental in metabolic diseases (Geng and Guo, 2024; Liu et al., 2021; Toledo et al., 2020). However, emerging evidence has revealed a protective role of SREBF1 under cellular stress conditions. In particular, SREBF1 has been reported to transcriptionally upregulate key anti-ferroptotic genes, such as GPX4 and SLC7A11, thereby enhancing cellular resistance to lipid peroxidation (Cao et al., 2024; Yi et al., 2020; Chen et al., 2025). Our findings strongly support this latter view, suggesting that in the context of AS-associated oxidative stress and iron dysregulation, the anti-ferroptotic function of SREBF1 may predominate. Downregulation of SREBF1 weakens cellular defense against ferroptosis, whereas sesamin restores SREBF1 expression *via* m^6^A-dependent regulation, resulting in upregulation of GPX4 and xCT and ultimately alleviating endothelial injury. These results yield new insight into the dual and context-dependent roles of SREBF1 in cardiovascular diseases.

To further confirm that SREBF1 is an indispensable downstream effector of sesamin, we performed rigorous functional rescue experiments, including METTL3/SREBF1 double knockdown and treatment with the ferroptosis inhibitor Ferrostatin-1. Notably, SREBF1 silencing attenuated the protective effects of sesamin on iron accumulation, lipid peroxidation, and oxidative stress, whereas concurrent METTL3 knockdown partially restored these protective effects. Similarly, Ferrostatin-1 treatment mimicked the endothelial protective effects of sesamin, confirming that ferroptosis inhibition is a critical component of sesamin-mediated cytoprotection. These observations indicate that the favorable effects of METTL3 inhibition are not mediated exclusively through SREBF1, consistent with the global regulatory nature of m^6^A modification. In cancer studies, METTL3 has been shown to modulate ferroptosis sensitivity by targeting multiple mRNA substrates simultaneously (Sun et al., 2023; Zeng et al., 2020). Accordingly, in the context of AS, sesamin-mediated METTL3 inhibition may stabilize additional, as yet unidentified, anti-ferroptosis–related mRNAs beyond SREBF1, collectively contributing to endothelial protection. This multi-target characteristic is a common feature of natural compounds and may partly explain their therapeutic efficacy. Our findings therefore highlight the complexity of sesamin’s mechanism of action and provide a rationale for future studies aimed at delineating its broader regulatory network.

Nevertheless, some limitations of this study should be acknowledged. First, whether sesamin directly binds to METTL3 and inhibits its enzymatic activity requires further confirmation using techniques such as surface plasmon resonance or isothermal titration calorimetry. Second, while our *in vitro* experiments using HUVECs support the involvement of endothelial cells, the *in vivo* data did not include endothelial cell-specific co-localization (e.g., SREBF1/METTL3 or ferroptosis markers with CD31 or VE-cadherin), and it remains unclear whether the METTL3/SREBF1-mediated mechanism operates in other AS-relevant cell types, such as macrophages or vascular smooth muscle cells. Future studies incorporating endothelial marker-based co-localization and investigating additional cell types are warranted. Third, as a transcription factor, the downstream gene network regulated by SREBF1, particularly genes involved in ferroptosis, has yet to be systematically identified and validated using genome-wide approaches such as ChIP-seq.

## Conclusion

In conclusion, this study reveals that sesamin alleviates ferroptosis-related endothelial injury by modulating the METTL3/SREBF1 axis. Supplementary experiments with Ferrostatin-1 and METTL3/SREBF1 double knockdown confirmed the critical role of ferroptosis and this regulatory pathway in sesamin-mediated endothelial protection. These findings provide mechanistic support for the anti-atherosclerotic effects of the Yishen Huoxue Huatan formula and highlight potential therapeutic strategies targeting m^6^A-dependent regulation and ferroptosis in atherosclerosis.

## Data Availability

The original contributions presented in the study are included in the article/Supplementary Material, further inquiries can be directed to the corresponding author.
