# Supplementary material for: Sesamin attenuates atherosclerosis by alleviating vascular endothelial ferroptosis-related injury via m6A-dependent regulation of SREBF1 expression
Source: Front Cell Dev Biol. 2026 Jun 23;14:1807359. doi: 10.3389/fcell.2026.1807359 (PMC13337896; doi:10.3389/fcell.2026.1807359)
Supplement: Supplementary file 1 [file Table1.docx]

Supplementary Table 1. Representative KEGG enrichment terms related to cell fate regulation and RNA-associated post-transcriptional regulatory mechanisms

| **Category** | **Subcategory** | **KEGG ID** | **Pathway** | **GeneRatio** | **Fold enrichment** | **Adjusted P value** | **Genes** | **Count** |
| --- | --- | --- | --- | --- | --- | --- | --- | --- |
| Cellular Processes | Cell growth and death | hsa04218 | Cellular senescence | 11/73 | 9.07 | 3.34 × 10⁻⁷ | 207/1026/3576/3486/3552/3569/2475/5970/7040/7157/5054 | 11 |
| Human Diseases | Cancer: overview | hsa05206 | MicroRNAs in cancer | 7/73 | 2.83 | 2.28 × 10⁻² | 1026/3162/4318/2475/5328/5743/7157 | 7 |
