# Supplementary figures and images for: Sesamin attenuates atherosclerosis by alleviating vascular endothelial ferroptosis-related injury via m6A-dependent regulation of SREBF1 expression
Source: Front Cell Dev Biol. 2026 Jun 23;14:1807359. doi: 10.3389/fcell.2026.1807359 (PMC13337896; doi:10.3389/fcell.2026.1807359)

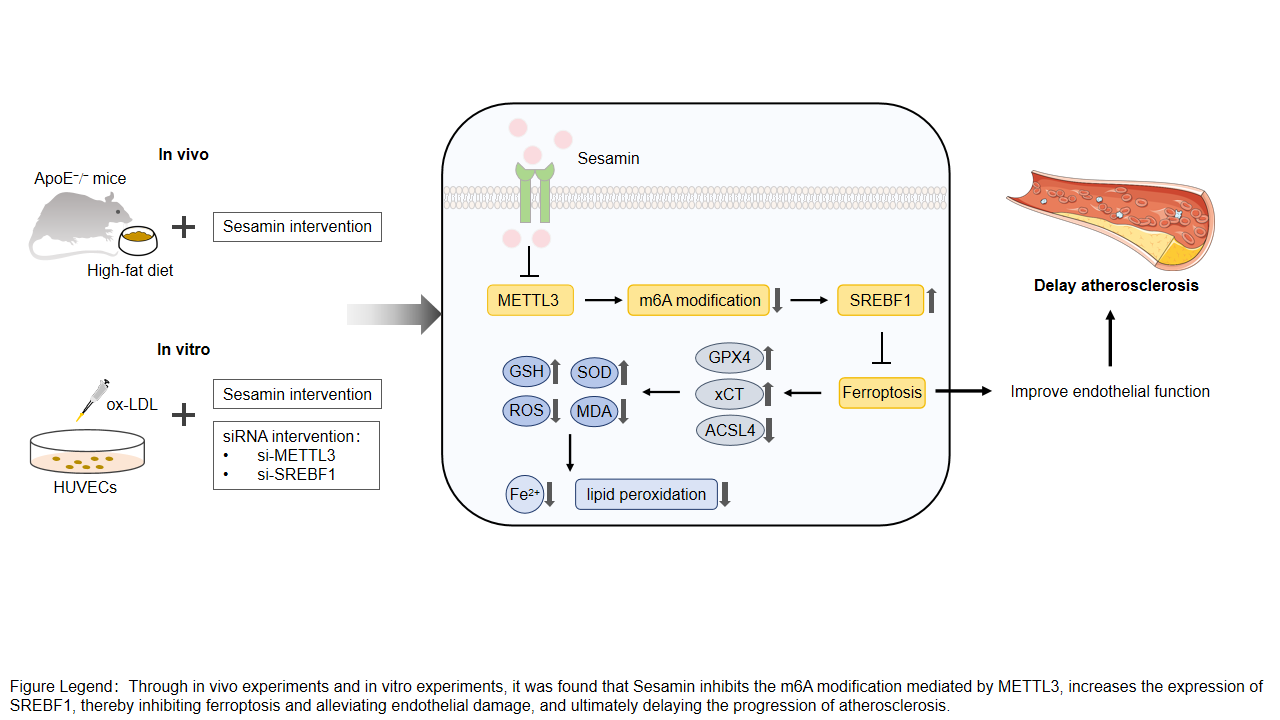

Supplement: Supplementary file 2 [file Image1.tif]
