# Supplementary material for: Sesamin attenuates atherosclerosis by alleviating vascular endothelial ferroptosis-related injury via m6A-dependent regulation of SREBF1 expression
Source: Front Cell Dev Biol. 2026 Jun 23;14:1807359. doi: 10.3389/fcell.2026.1807359 (PMC13337896; doi:10.3389/fcell.2026.1807359)
Supplement: Supplementary file 3 [file DataSheet1.pdf]

Figure 3D

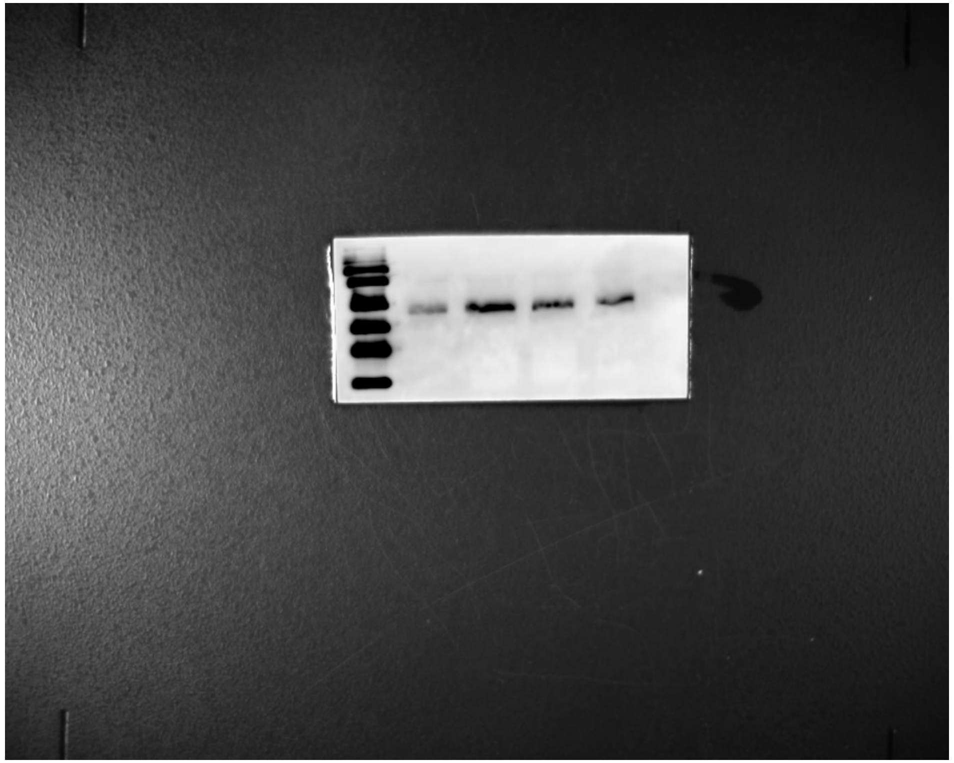

The original Western Blot images of ACSL4 in Figure 3D. From left to right: Ctrl, AS, AS+Sesamin-50, AS+Sesamin-50.

Figure 3D

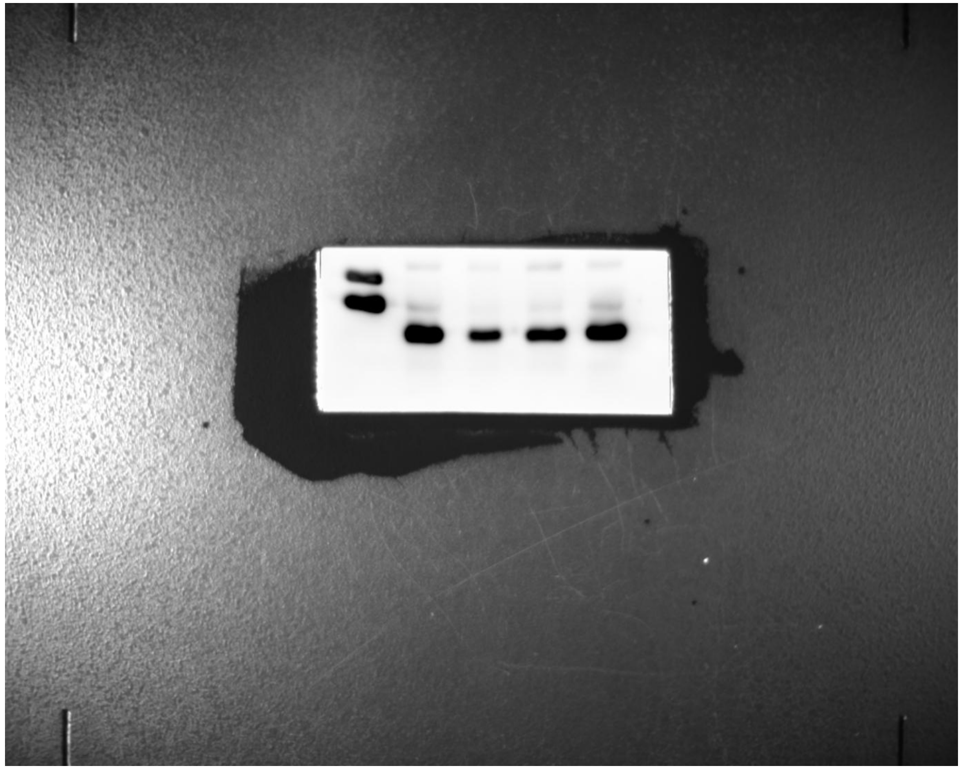

The original Western Blot images of GPX4 p65 in Figure 3D. From left to right: Ctrl, AS, AS+Sesamin-50, AS+Sesamin-50.

Figure 3D

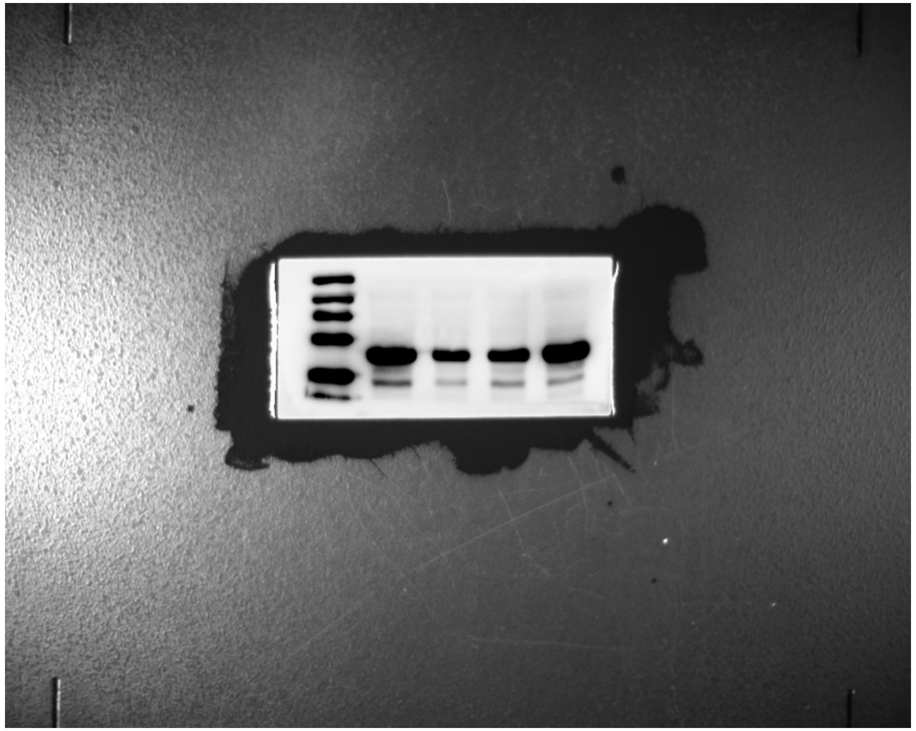

The original Western Blot images of xCT in Figure 3D. From left to right: Ctrl, AS, AS+Sesamin-50, AS+Sesamin-50.

Figure 3D

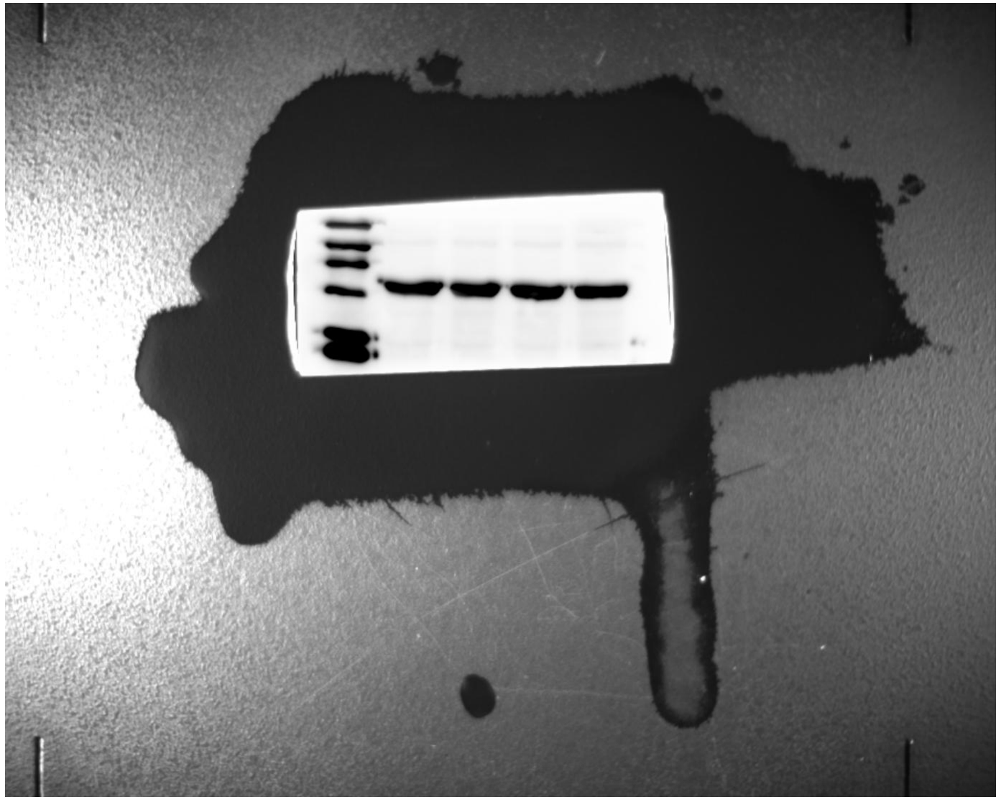

The original Western Blot images of β-actin in Figure 3D. From left to right: Ctrl, AS, AS+Sesamin-50, AS+Sesamin-50.

Figure S2B

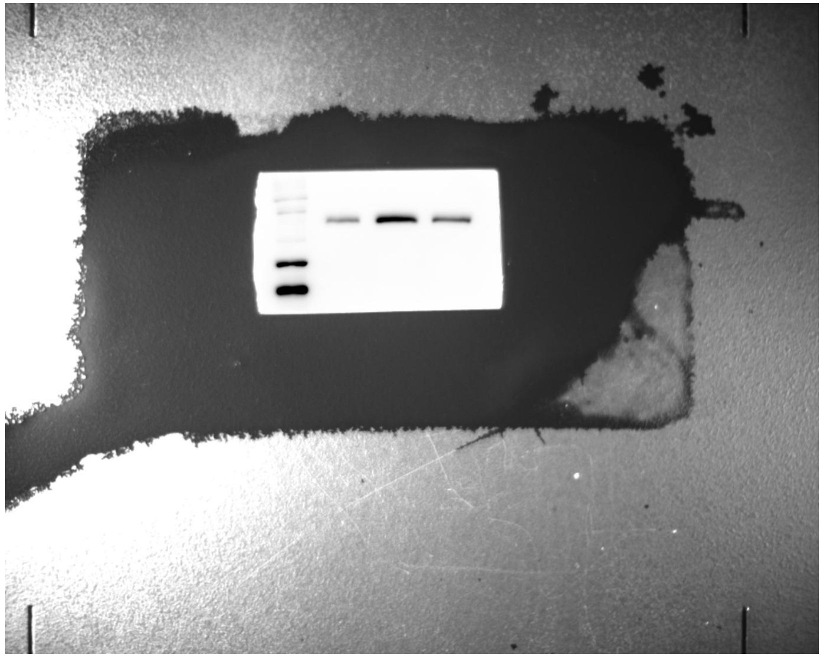

The original Western Blot images of β-actin in Figure S2B. From left to right: Control, ox-LDL 100μg/mL, ox-LDL 100μg/mL+Sesamin 50μM.

Figure S2B

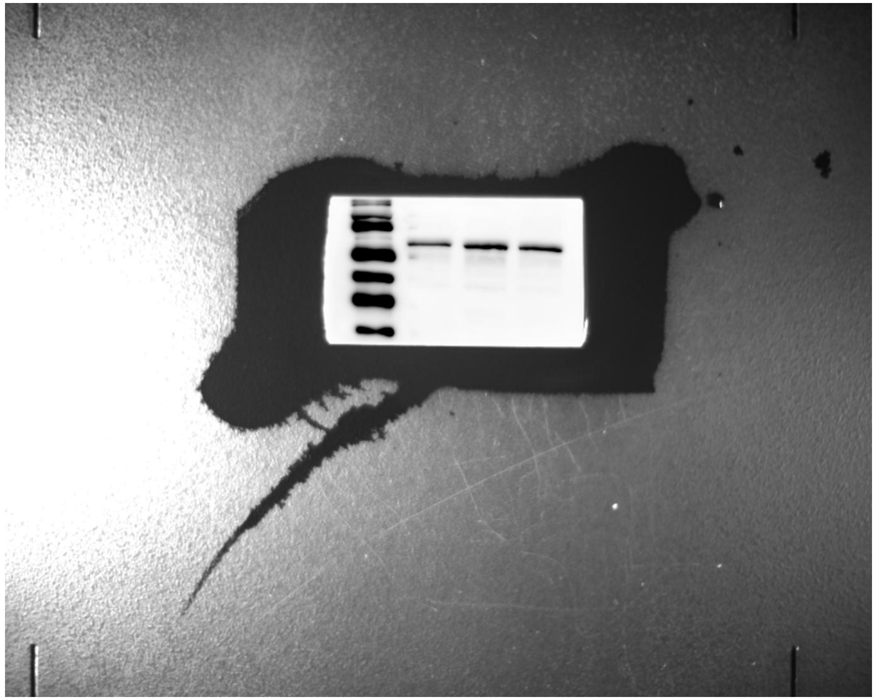

The original Western Blot images of β-actin in Figure S2B. From left to right: Control, ox-LDL 100μg/mL, ox-LDL 100μg/mL+Sesamin 50μM.

Figure S2B

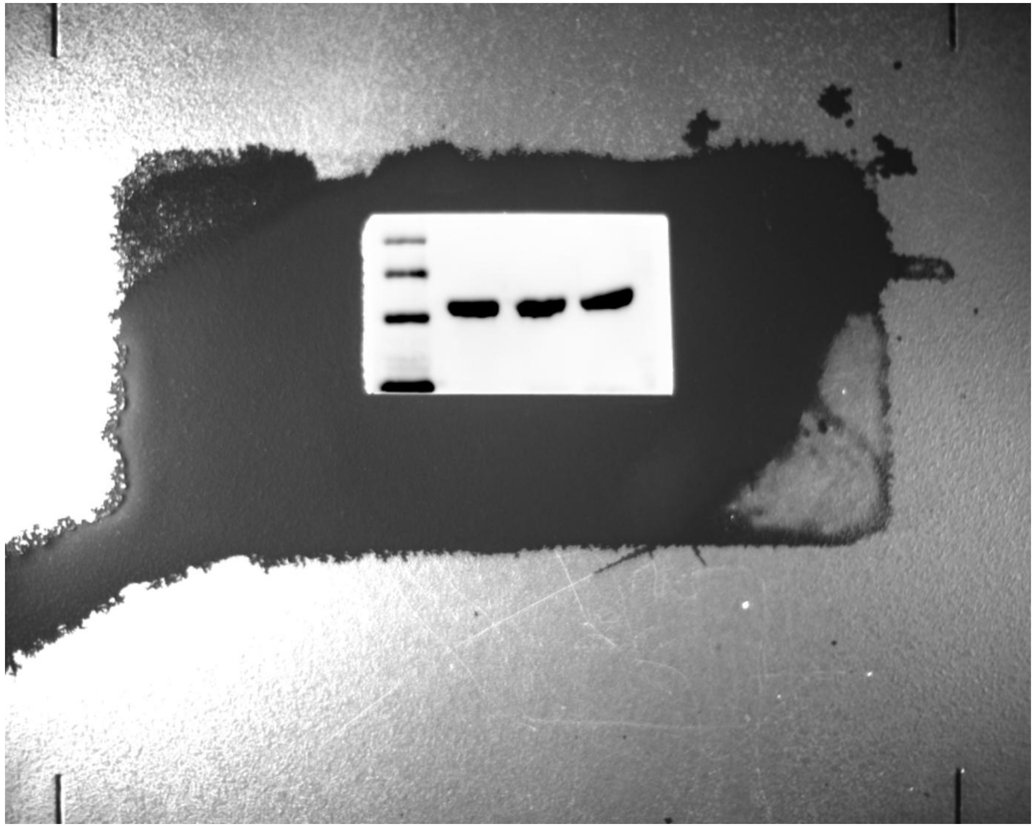

The original Western Blot images of β-actin in Figure S2B. From left to right: Control, ox-LDL 100μg/mL, ox-LDL 100μg/mL+Sesamin 50μM.

Figure 4H

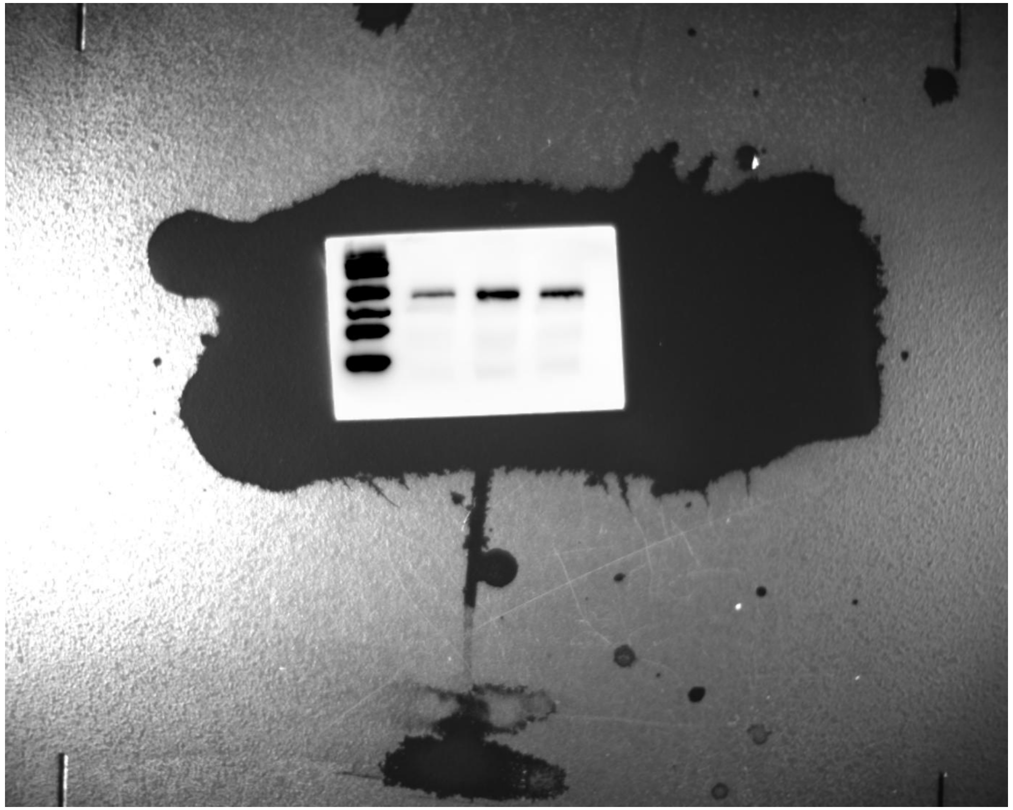

The original Western Blot images of ACSL4 in Figure 4H. From left to right: Control, ox-LDL, ox-LDL + sesamin.

Figure 4H

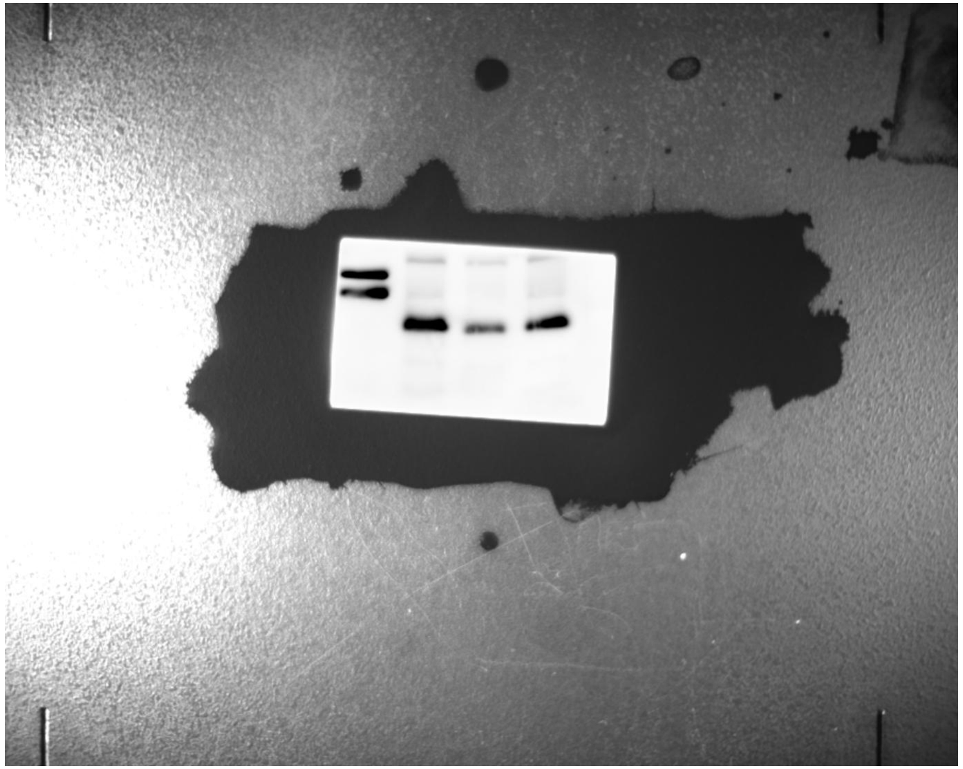

The original Western Blot images of GPX4 in Figure 4H. From left to right: Control, ox-LDL, ox-LDL + sesamin.

Figure 4H

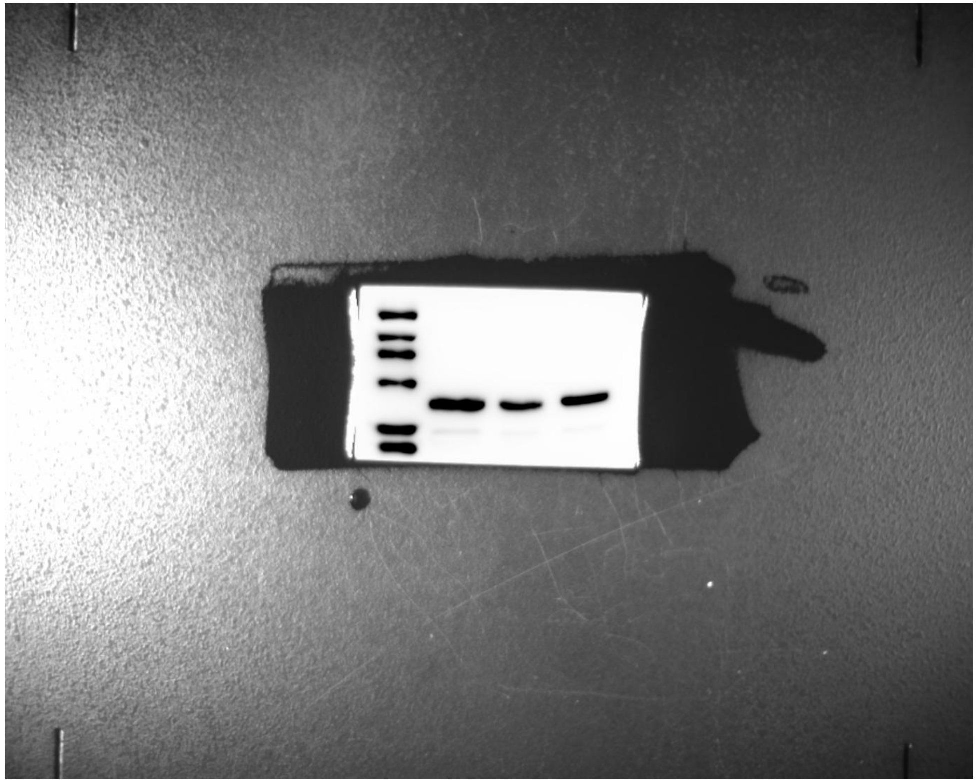

The original Western Blot images of xCT in Figure 4H. From left to right: Control, ox-LDL, ox-LDL + sesamin.

Figure 4H

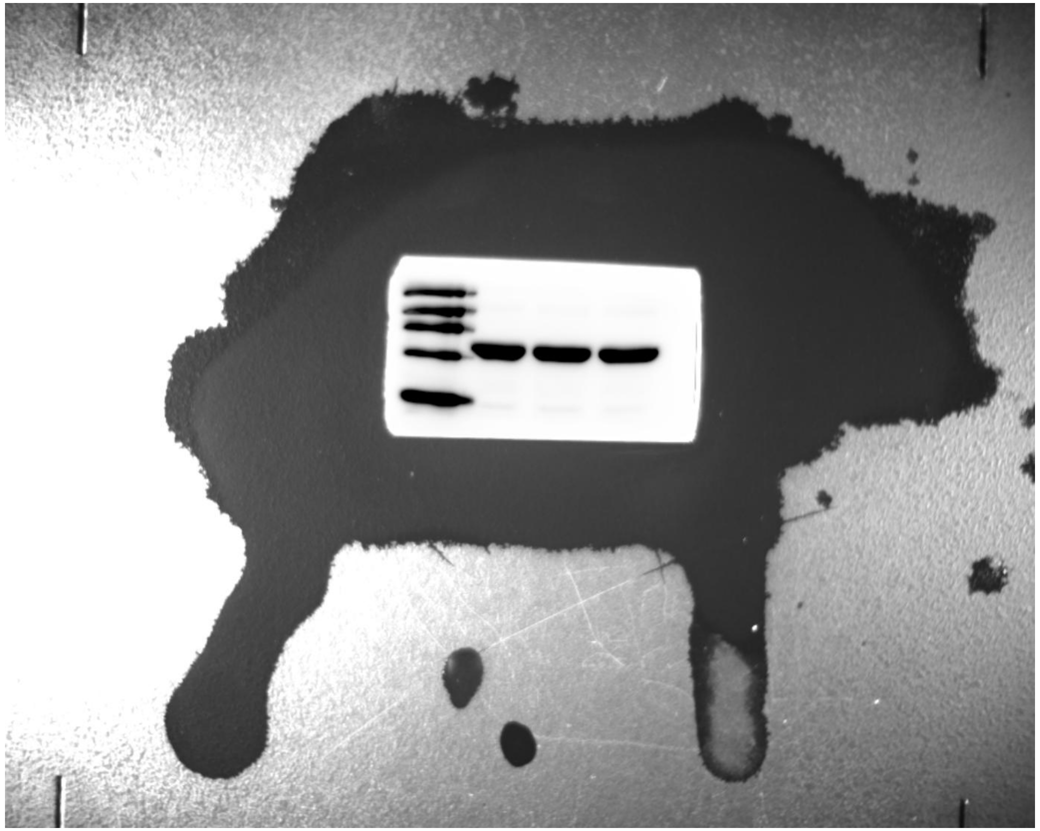

The original Western Blot images of β-actin in Figure 4H. From left to right: Control, ox-LDL, ox-LDL + sesamin.

Figure S3B

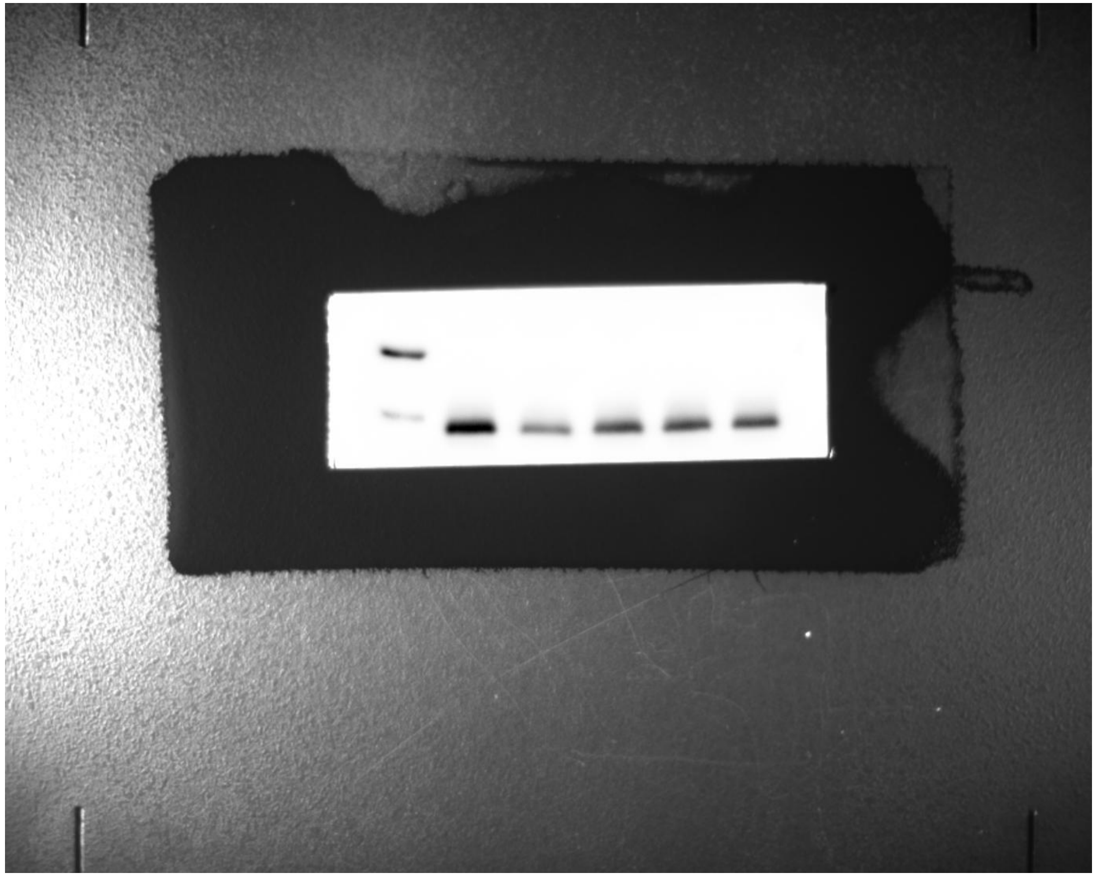

The original Western Blot images of β-actin in Figure S3B. From left to right: Control, ox-LDL, ox-LDL + sesamin， ox-LDL+Fer-1， ox-LDL+Sesamin+Fer-1.

Figure S3B

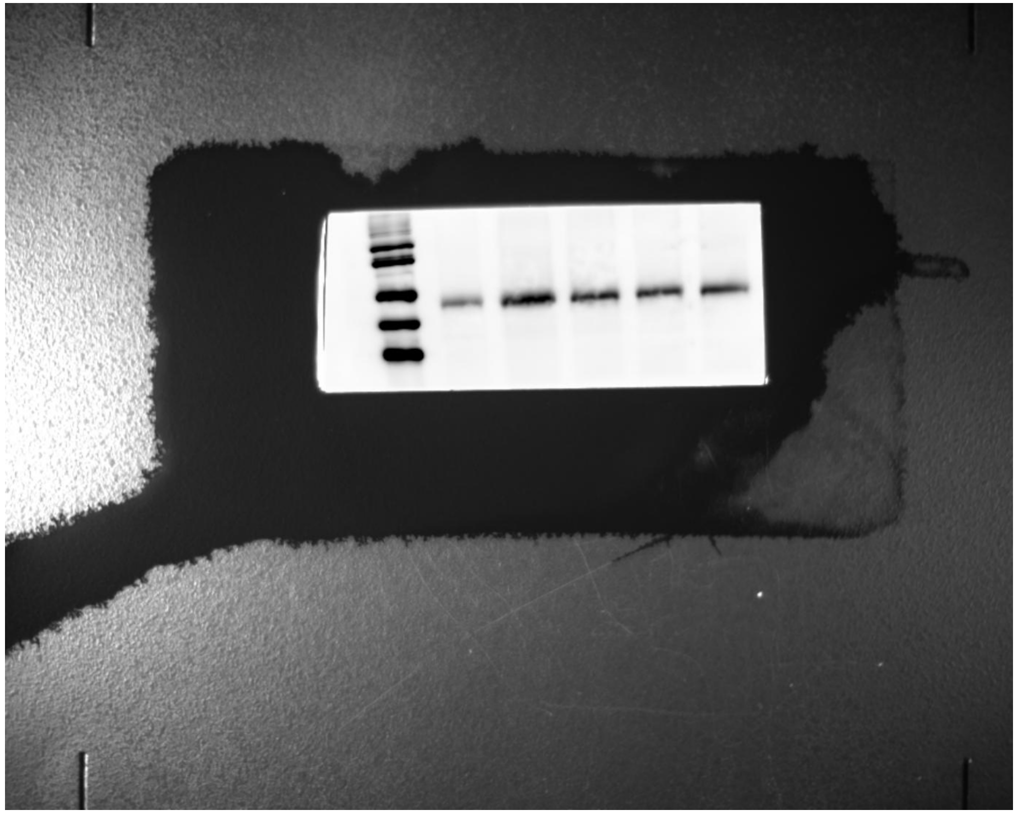

The original Western Blot images of β-actin in Figure S3B. From left to right: Control, ox-LDL, ox-LDL + sesamin， ox-LDL+Fer-1， ox-LDL+Sesamin+Fer-1.

Figure S3B

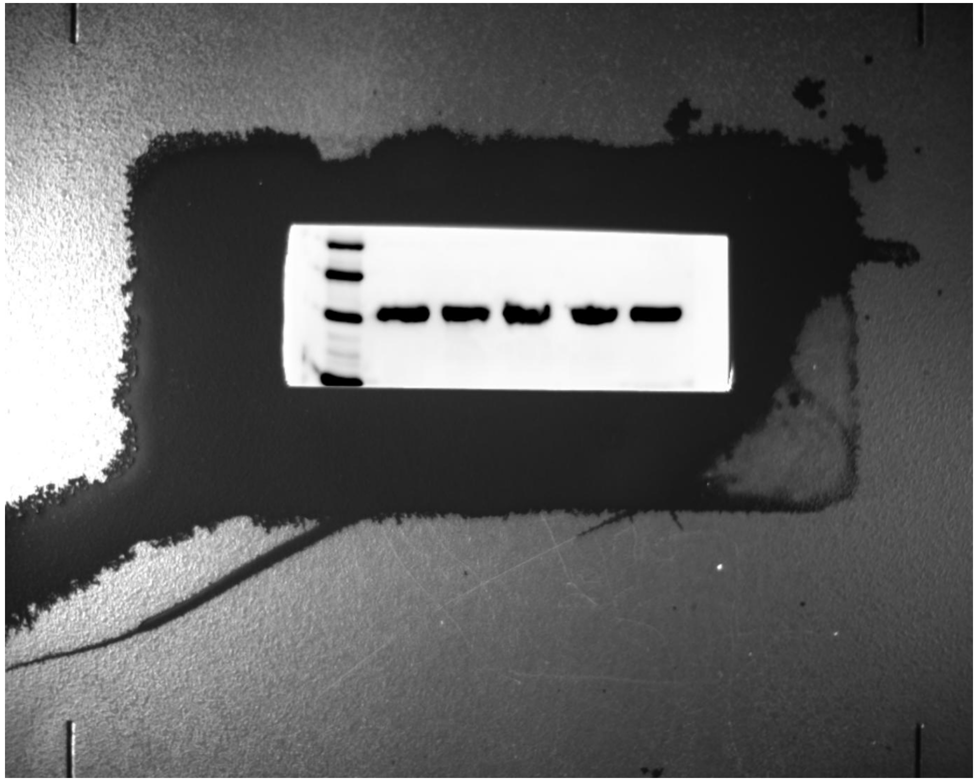

The original Western Blot images of β-actin in Figure S3B. From left to right: Control, ox-LDL, ox-LDL + sesamin， ox-LDL+Fer-1， ox-LDL+Sesamin+Fer-1.

Figure 5C

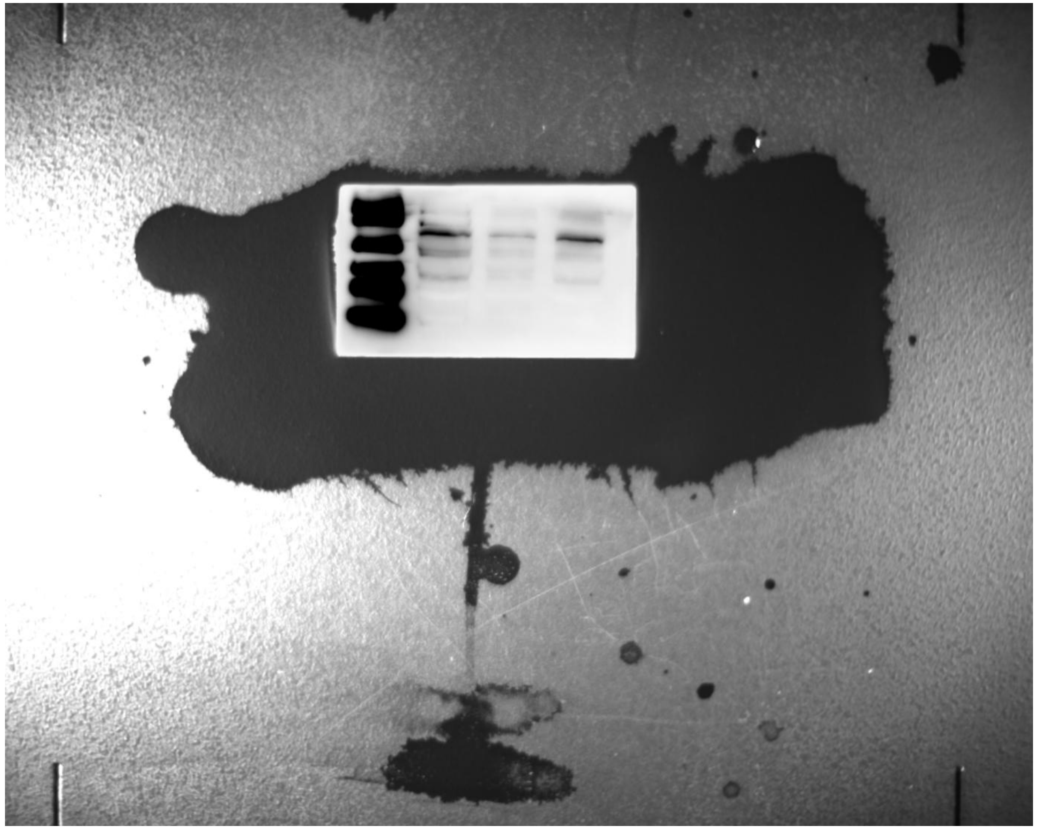

The original Western Blot images of SREBF1 in Figure 5C. From left to right: Control, ox-LDL, ox-LDL + sesamin.

Figure 5C

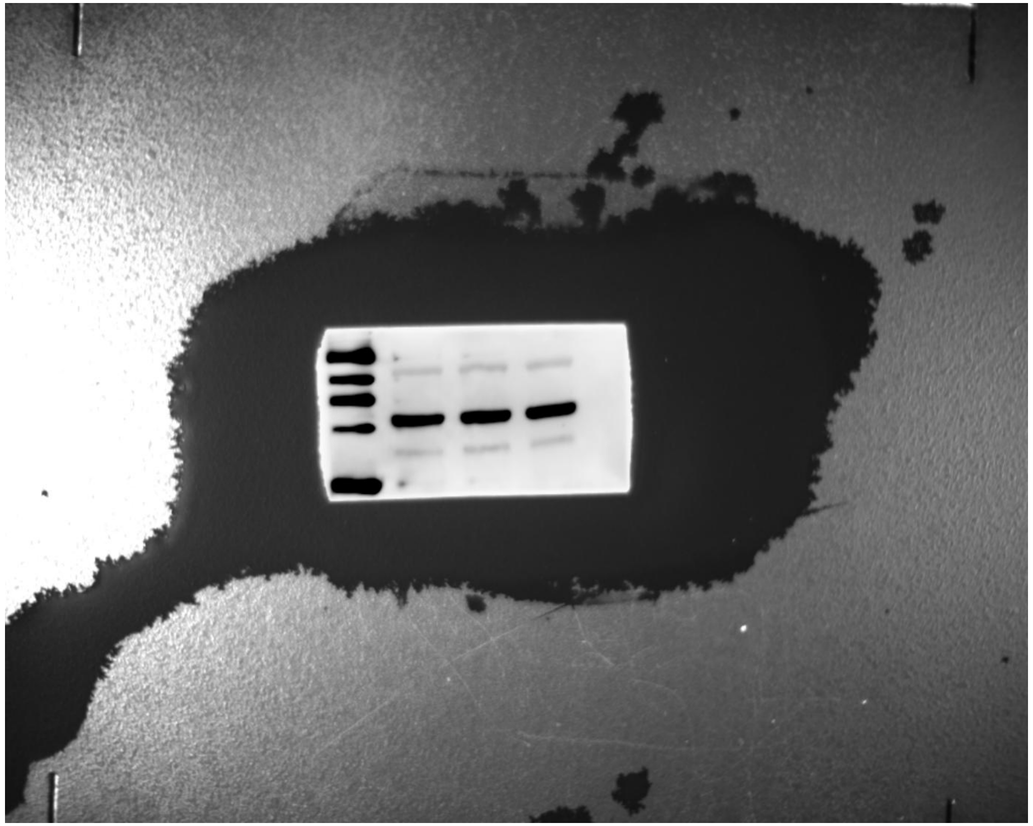

The original Western Blot images of β-actin in Figure 5C. From left to right: Control, ox-LDL, ox-LDL + sesamin.

Figure 5F

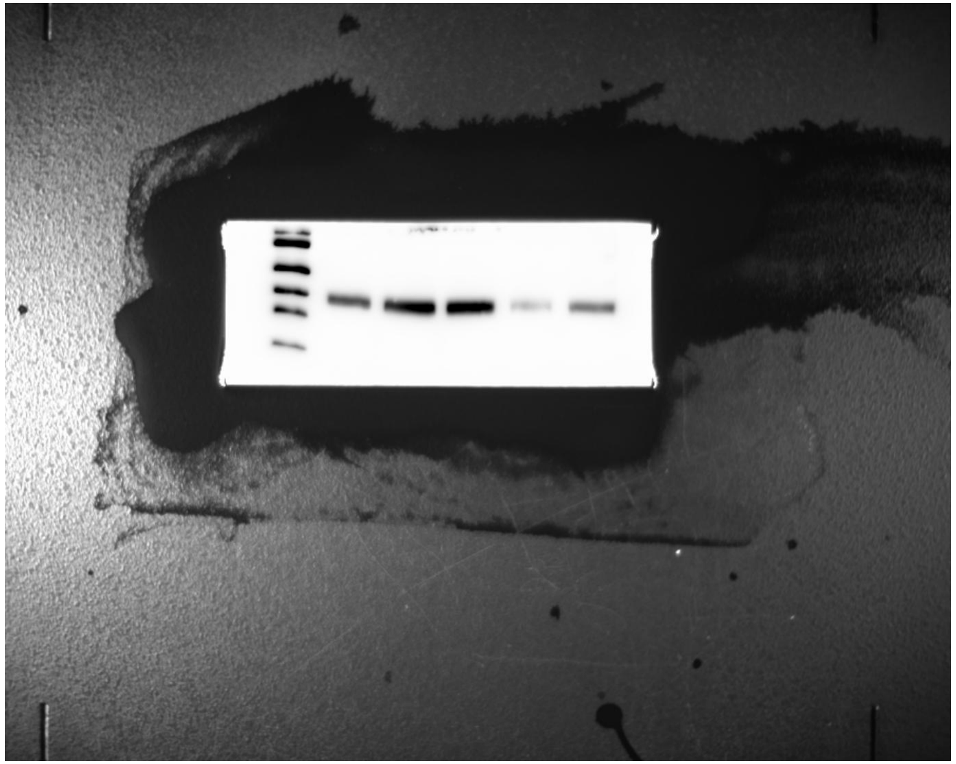

The original Western Blot images of METTL3 in Figure 5F. From left to right: control, ox-LDL, ox-LDL + si-NC, ox-LDL + si-METTL3, and ox-LDL + sesamin groups.

Figure 5F

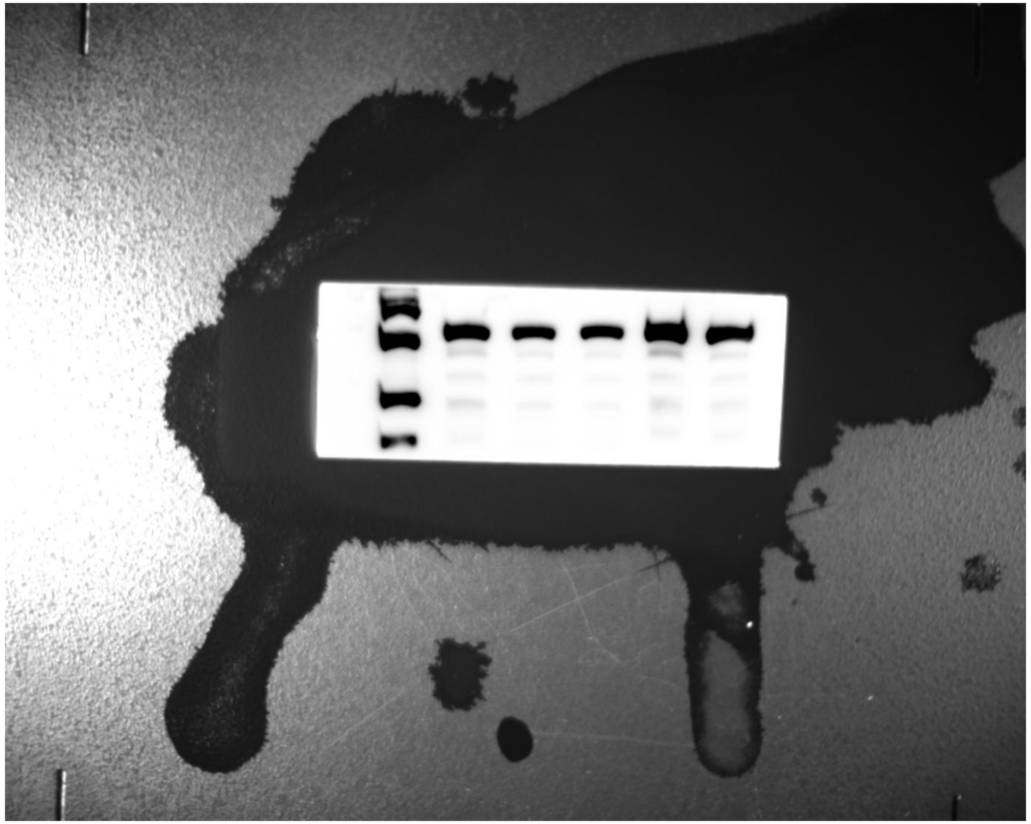

The original Western Blot images of SREBF1 in Figure 5F. From left to right: control, ox-LDL, ox-LDL + si-NC, ox-LDL + si-METTL3, and ox-LDL + sesamin groups.

Figure 5F

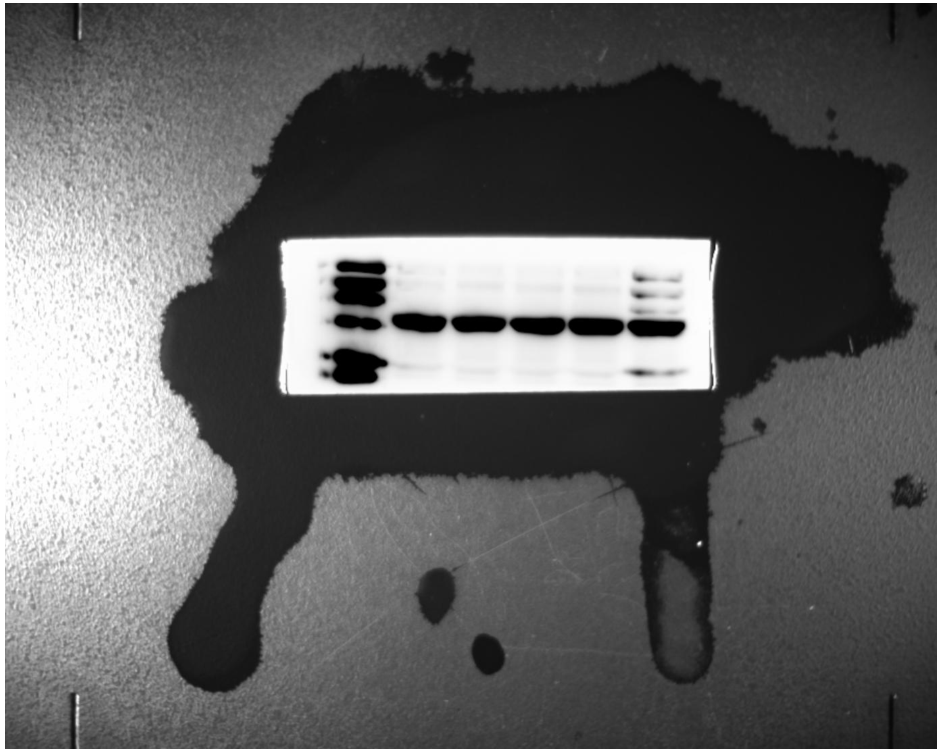

The original Western Blot images of β-actin in Figure 5F. From left to right: control, ox-LDL, ox-LDL + si-NC, ox-LDL + si-METTL3, and ox-LDL + sesamin groups.

Figure 6F

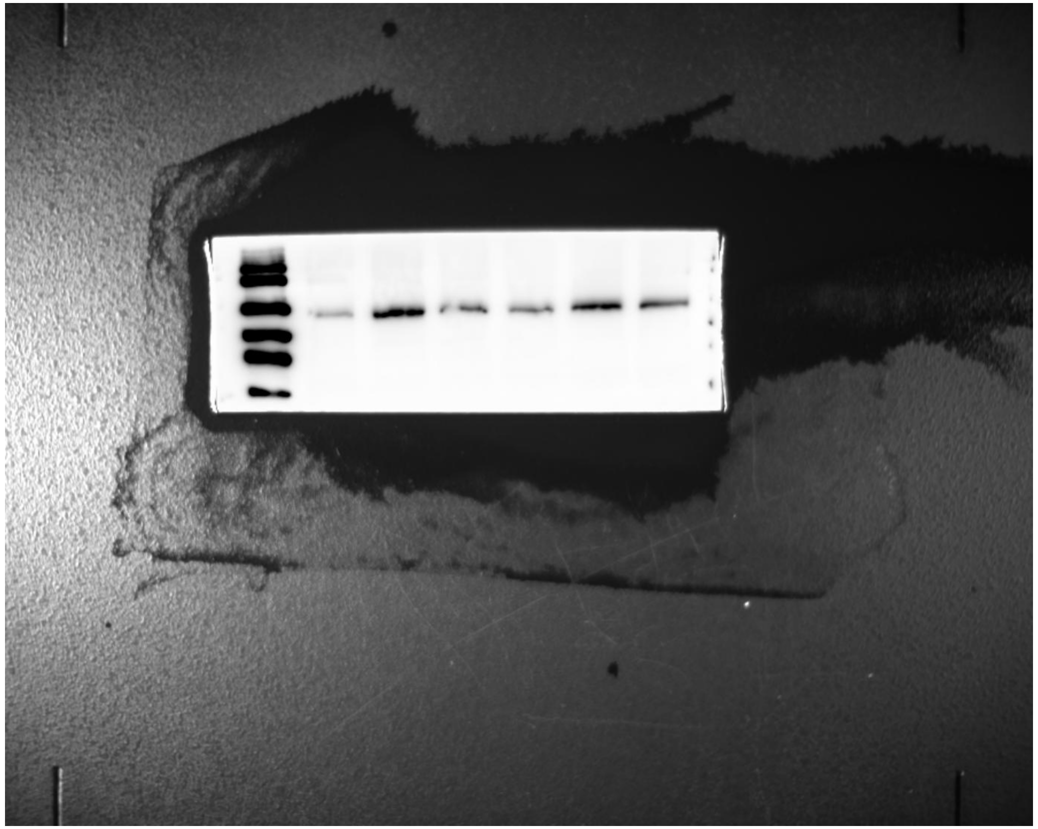

The original Western Blot images of ACSL4 in Figure 6F. From left to right: Control, ox-LDL, ox-LDL + sesamin, ox-LDL + sesamin + si-NC, ox-LDL + sesamin + si-SREBF1, ox-LDL + sesamin + si-SREBF1 + si-METTL3 groups.

Figure 6F

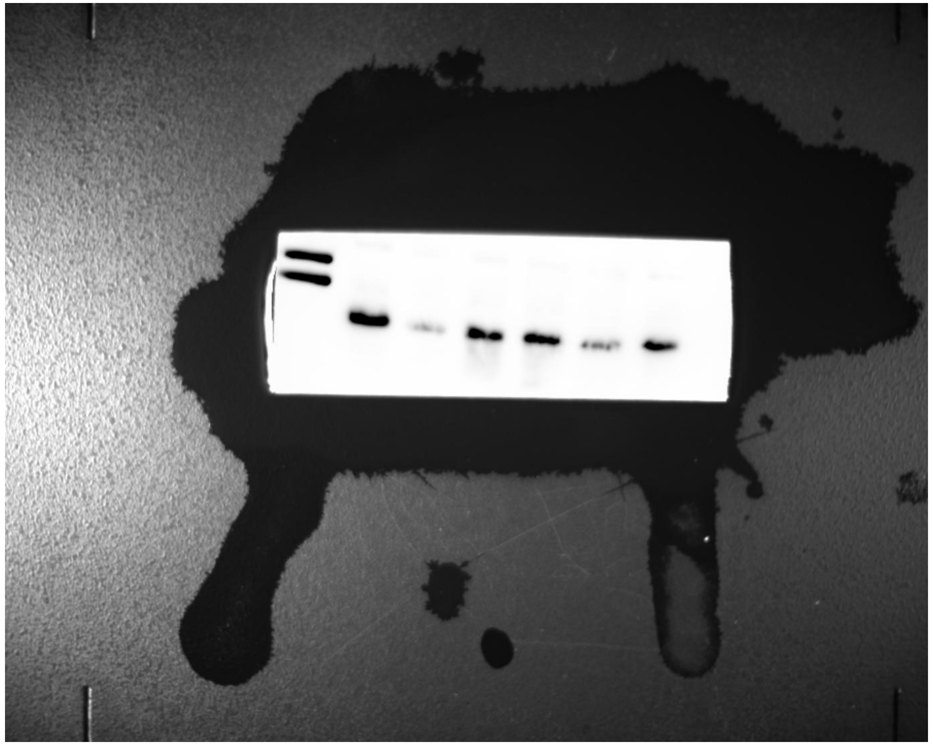

The original Western Blot images of GPX4 in Figure 6F. From left to right: Control, ox-LDL, ox-LDL + sesamin, ox-LDL + sesamin + si-NC, ox-LDL + sesamin + si-SREBF1, ox-LDL + sesamin + si-SREBF1 + si-METTL3 groups..

Figure 6F

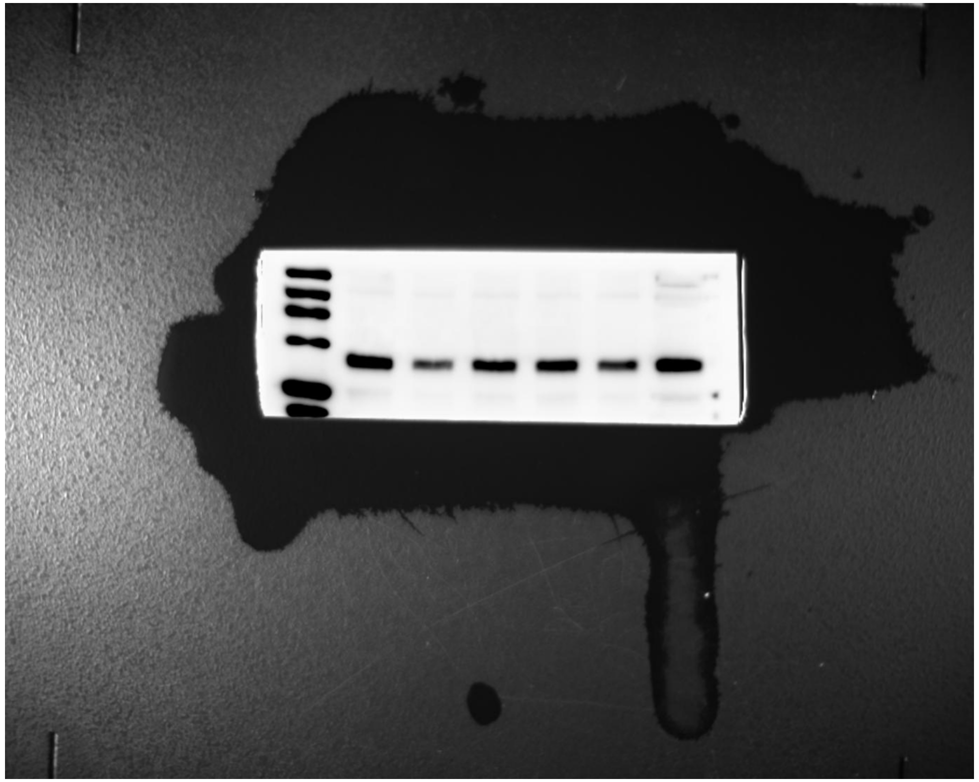

The original Western Blot images of xCT in Figure 6F. From left to right: Control, ox-LDL, ox-LDL + sesamin, ox-LDL + sesamin + si-NC, ox-LDL + sesamin + si-SREBF1, ox-LDL + sesamin + si-SREBF1 + si-METTL3 groups..

Figure 6F

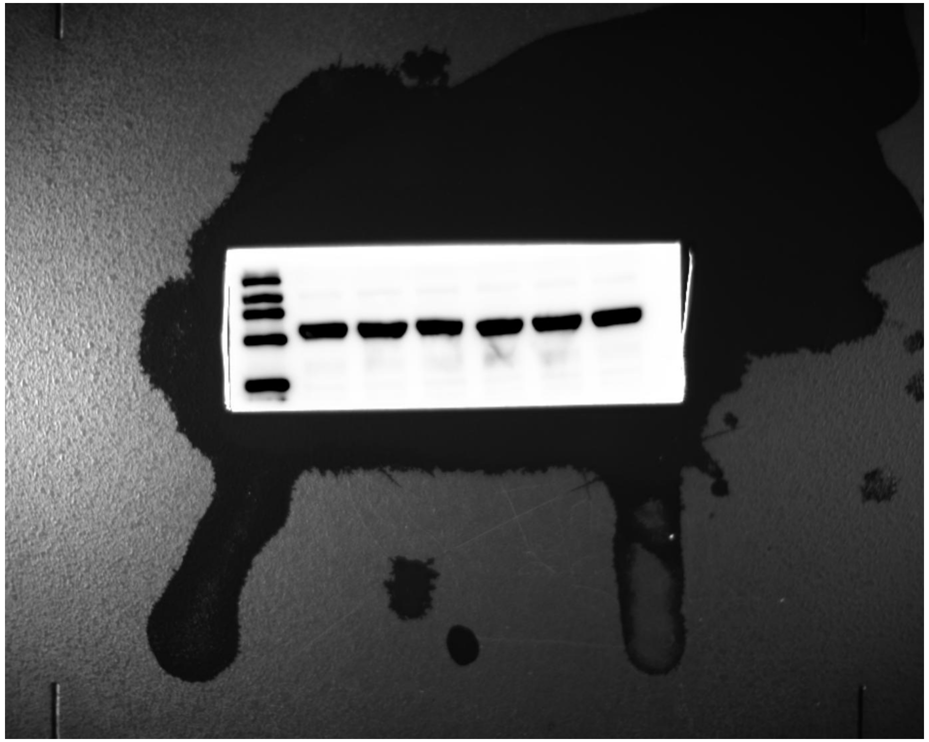

The original Western Blot images of β-actin in Figure 6F. From left to right: Control, ox-LDL, ox-LDL + sesamin, ox-LDL + sesamin + si-NC, ox-LDL + sesamin + si-SREBF1, ox-LDL + sesamin + si-SREBF1 + si-METTL3 groups.
